# A Nanos3-containing protein complex can activate RNA translation in primordial germ cells in vivo

**DOI:** 10.1038/s44319-026-00781-w

**Published:** 2026-04-24

**Authors:** Antra Gupta, Arzu Melsa Basaran, Moritz Ophaus, Shantanu Madiwale, Martin Stehling, Michal Reichman-Fried, Katsiaryna Tarbashevich, Erez Raz

**Affiliations:** 1 Institute of Cell Biology, Center for Molecular Biology of Inflammation (ZMBE), University of Münster, Münster, Germany; 2 Max Planck Institute for Molecular Biomedicine, Münster, Germany

**Keywords:** Development, RNA Biology, Translation & Protein Quality

## Abstract

Maintaining the germline fate requires tight post-transcriptional control of RNA function. Here, we investigate how primordial germ cell (PGC) identity is maintained in zebrafish and reveal that the conserved RNA-binding proteins Nanos3 and Dead End1 form a complex that safeguards PGC identity. Using transcriptomics and in vivo imaging-based analyses, we show that this complex controls the translational activation and localization of both *nanos3* and *dead end1* RNAs, establishing a positive feedback loop crucial for regulating their protein expression. These findings uncover a previously unknown layer of control over germline development, where a complex containing Nanos3, a protein associated with the inhibition of RNA translation, acts as an activator by interacting with an eIF3 complex protein to promote translation, thereby maintaining specific RNAs at the periphery of phase-separated germ cell granules. Disrupting the physical interaction between Nanos3 and Dead End1 leads to transdifferentiation of germ cells into somatic lineages. Overall, our findings identify a self-sustaining mechanism of translational activation in vivo, positioning the Nanos3–Dead End1 complex as a central effector of germline fate.

## Synopsis

Nanos3 and Dnd1 form a complex that can bind the 3′UTRs of target RNAs and promote their translation in zebrafish primordial germ cells. This reveals a role for Nanos3 in translational activation in vivo by coupling RNA binding at the 3′UTR to the translation initiation machinery.

- Nanos3 and Dnd1 form a complex that is required to maintain primordial germ cell fate in vivo.
- The complex interacts with the translation initiation machinery via eIF3d.
- Nanos3 and Dnd1 bind the 3’UTR of target RNAs to promote their translation and maintenance at the germ granule periphery.

## Abstract (web summary)

Nanos3 and Dnd1 form a complex that can bind the 3′UTRs of target RNAs and promote their translation in zebrafish primordial germ cells. This reveals a role for Nanos3 in translational activation in vivo by coupling RNA binding at the 3′UTR to the translation initiation machinery.

## Graphical abstract

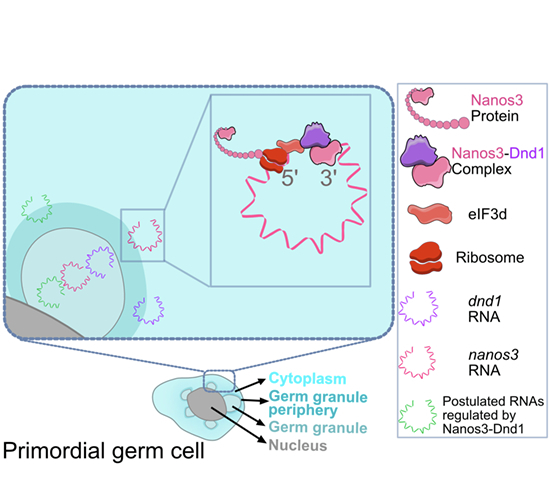

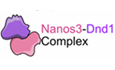

## Introduction

Primordial germ cells (PGCs) are specified early in embryogenesis and require tightly regulated molecular mechanisms to establish and maintain their fate, thereby ensuring the transmission of genetic information across generations (Chiquoine, 1954; Wylie, 1999; Raz, 2002; Strome and Updike, 2015; Chen et al, 2025). Indeed, defects in PGC development result in infertility and are also associated with the formation of germ cell tumors (Youngren et al, 2005; Nicholls and Page, 2021; Ramalingam, 2023; Westerich et al, 2023a; Tüttelmann et al, 2025). Among the regulatory factors involved, RNA-binding proteins (RBPs) play key roles in PGC development by regulating specification, maintaining germ cell identity, and directing their differentiation into functional gametes through the translational control of specific RNAs (Crittenden et al, 2002; Albarqi and Ryder, 2023; Colegrove-Otero et al, 2005; Besse and Ephrussi, 2008).

The evolutionarily conserved Nanos family of RBPs plays key roles in germ cell development across a broad range of invertebrate and vertebrate species (Wang and Lehmann, 1991; Kobayashi et al, 1996; Subramaniam and Seydoux, 1999; Köprunner et al, 2001; Tsuda et al, 2003; Julaton and Reijo Pera, 2011). The zebrafish Nanos3 ortholog is expressed in PGCs from the time they are specified and continues to function later in germline development to sustain PGC development and oocyte production (Köprunner et al, 2001; Draper et al, 2007; referred to as Nanos1 in these studies; Westerich et al, 2023b). Importantly, in medaka, ectopic co-expression of Nanos3 and Dead End1 (Dnd1) in somatic cells induces the formation of PGC-like cells that successfully migrate to the gonads and differentiate into functional gametes (Nishimura and Fujimoto, 2025), underscoring Nanos3 as a central regulator of germ cell fate. At the molecular level, members of the Nanos protein family have been shown to repress RNA translation, often in cooperation with the RBP Pumilio in *Drosophila* (Murata and Wharton, 1995; Forbes and Lehmann, 1998; Kraemer et al, 1999; Jaruzelska et al, 2003). While the role of Nanos proteins as translational repressors is well established, their potential function as positive regulators of translation in vivo has not been reported on.

Studies aimed at understanding Nanos3 function in in vitro systems have shown that Nanos family proteins interact with Dead End1 (DND1), a key regulator of germ cell fate in vertebrates (Weidinger et al, 2003; Gross-Thebing et al, 2017; Hirano et al, 2022; Westerich et al, 2023b; Wang et al, 2025). As also shown in vitro, the NANOS2–DND1 complex interacts with the CCR4–NOT deadenylase complex, thereby promoting mRNA degradation and translational repression of specific target transcripts (Hirano et al, 2022; Suzuki et al, 2016).

Dnd1 has emerged as a versatile post-transcriptional regulator that promotes RNA translation and stability in various contexts. In vivo findings from zebrafish and *Xenopus* suggest that Dnd1 enables the translation of specific RNAs critical for germ cell development. In zebrafish, Dnd1 binds the 3’ untranslated region (UTR) of *nanos3* RNA, thereby ensuring Nanos3 protein production (Kedde et al, 2007). Similarly, to activate *nanos* RNA translation in *Xenopus*, Dnd1 modulates the function of eIF3f (Aguero et al, 2017). Lastly, in vitro studies have shown that Dnd1 can also stabilize transcripts encoding tumor suppressors such as *LATS2* and *p27*, highlighting its broader role in suppressing tumor development and exerting post-transcriptional control (Kedde et al, 2007). Yet, despite the central role of Dnd1 in germline development, remarkably little is known about the mechanisms governing its own RNA expression, stability, and translation.

Motivated by the observations presented above, here we investigate the function of Nanos3 and Dnd1 in vivo in the context of the developing zebrafish embryo. Here, we show that zebrafish Nanos3 and Dnd1 form a functional complex in live PGCs and act cooperatively to activate RNA translation and control its localization. We also show that both proteins are required for the localization and translation of their own RNAs as well as for each other’s, revealing an interdependent positive regulatory loop. Lastly, we identify an association between Dnd1 and the translation initiation machinery, providing mechanistic insight into how this complex modulates protein synthesis. Together, these findings uncover a previously unknown function of the Nanos3 protein and the molecular mechanisms by which it acts together with Dnd1 to control RNA function and preserve PGC identity.

## Results

### Nanos3 maintains zebrafish primordial germ cell fate in vivo

**Figure 1:**
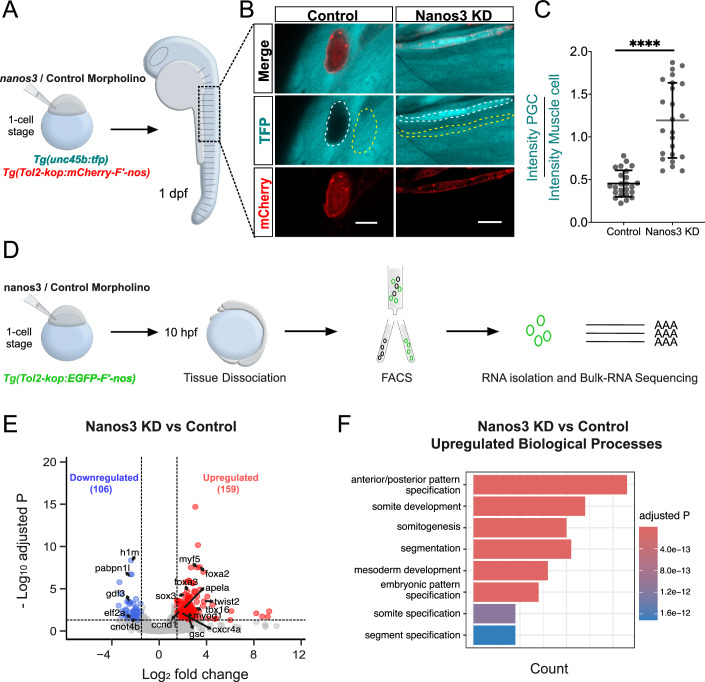
Nanos3 is required to preserve zebrafish primordial germ cell fate. (**A**) A schematic of the experimental setup. One-cell-stage embryos were obtained from *Tg [(unc45b: TFP)* x *(kop: mCherry-F*′*-nos 3’UTR)]* fish were injected either with a control or *nanos3* morpholino, along with a *cxcl12a* morpholino to induce ectopic positioning of primordial germ cells (PGCs) (e.g., within developing muscle). Imaging was performed at 1-day post-fertilization (dpf). (**B**) Confocal images showing PGCs (red, mCherry-F′) embedded within the muscle tissue (cyan, TFP) in control embryos (left) and Nanos3 knockdown (KD) (right) embryos. White dashed lines mark PGC boundaries. Yellow dashed lines mark a similar area within the neighboring somatic tissue. Scale bar: 10 µm. (**C**) Quantification of TFP fluorescence intensity presented as ratios between the signal level within the PGCs and that in the neighboring muscle tissue. Data represent mean ± SD (*n* = 24 cells per condition, N = 3 experiments). Mann–Whitney *U* test, ****P < 0.0001. (**D**) Workflow schematic for PGC transcriptomic profiling. Embryos from *Tg (kop: EGFP-F*′*- nos 3’UTR)* fish were dissociated at 10 h post-fertilization (hpf), EGFP-positive PGCs were isolated via fluorescence-activated cell sorting (FACS), and subjected to bulk RNA sequencing. (**E**) Volcano plot showing differential mRNA expression between control and Nanos3 KD PGCs. Blue: significantly downregulated transcripts; red: significantly upregulated transcripts. Differential expression is defined as adjusted *P* < 0.05 using DESeq2 (Wald test with Benjamini–Hochberg adjusted *P* values). The complete list of differentially expressed transcripts is provided in Dataset EV1. *n* = 3 independent biological replicates. (**F**) Gene Ontology (GO) enrichment analysis for transcripts upregulated in Nanos3 KD PGCs relative to control PGCs. Biological processes associated with somatic differentiation are prominently enriched. TFP teal fluorescent protein, F′ farnesylation signal, *nos 3’UTR* *nanos* 3′ untranslated region.

**Figure EV1:**
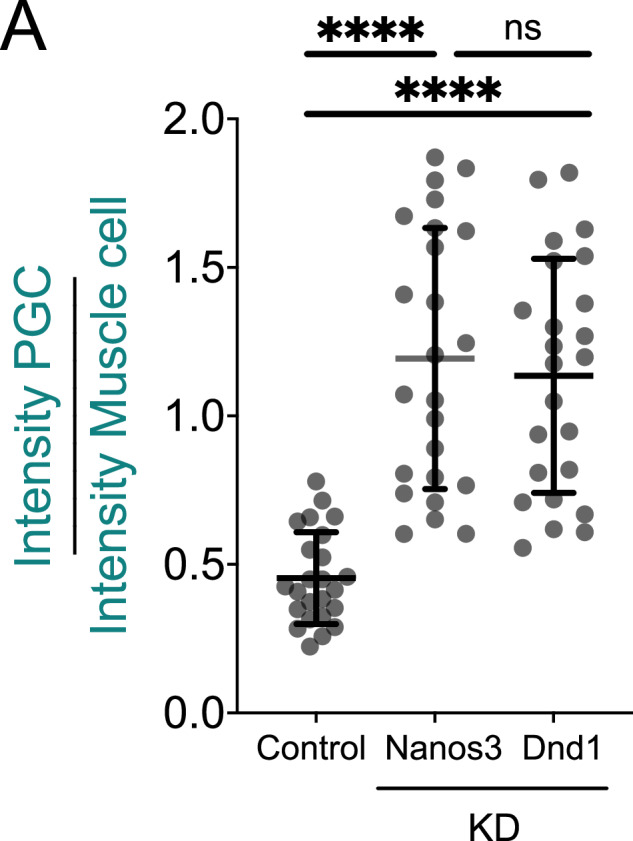
Similarity between Nanos3 and Dnd1 knockdown phenotypes. (**A**) Quantification of PGC-to-muscle TFP (teal fluorescent protein) fluorescence intensity ratios in Nanos3 knockdown (KD), Dnd1 KD, and control cells. See Fig. 1A,B for the experimental setup. Data represent mean ± SD (*n* = 20 cells per condition, *N* = 3 experiments). Mann–Whitney *U* test. ****P < 0.0001; ns not significant.

Zebrafish Nanos3 was originally shown to be crucial for the survival and migration of PGCs (Köprunner et al, 2001), and more recent studies based on cell morphology have suggested that it also plays a role in regulating germ cell fate (Westerich et al, 2023b). To investigate the molecular mechanisms by which Nanos3 modulates PGC development, we first examined the activity of the muscle-specific promoter *unc45b* (Etard et al, 2015) in PGCs positioned ectopically within the developing muscle tissue (Fig. 1A,B). Intriguingly, in these embryos, knockdown of Nanos3 (Nanos3 KD) using morpholino antisense oligonucleotides exhibited a strong elevation in expression of the fluorescent reporter (teal fluorescent protein, TFP) driven by the muscle-specific promoter (Fig. 1B,C), resembling the phenotype observed upon Dnd1 knockdown (Dnd1 KD) (Gross-Thebing et al, 2017) (for a direct comparison, see Fig. EV1A). These findings are consistent with the idea that both proteins act within a common molecular pathway to maintain PGC identity.

To obtain a more comprehensive view of Nanos3’s role in controlling PGC fate, we performed bulk RNA sequencing to compare the transcriptome of Nanos3 KD PGCs with that of control PGCs (Fig. 1D). Loss of Nanos3 resulted in upregulation of transcripts characteristic of somatic differentiation and a concomitant downregulation of germ cell-associated transcripts (Fig. 1E,F; Dataset EV1).

Together, the morphological changes observed in PGCs following Nanos3 depletion, as previously reported (Westerich et al, 2023b), combined with the transcriptomic changes we observed here, support the idea that the loss of Nanos3 drives PGCs toward somatic differentiation. The resemblance of this phenotype to that induced by Dnd1 KD (Gross-Thebing et al, 2017) suggests that Nanos3 functions together with Dnd1 as a key molecular regulator of PGC fate in a shared developmental pathway.

### Nanos3 and Dead End1 cooperatively regulate a similar set of RNA molecules

**Figure 2:**
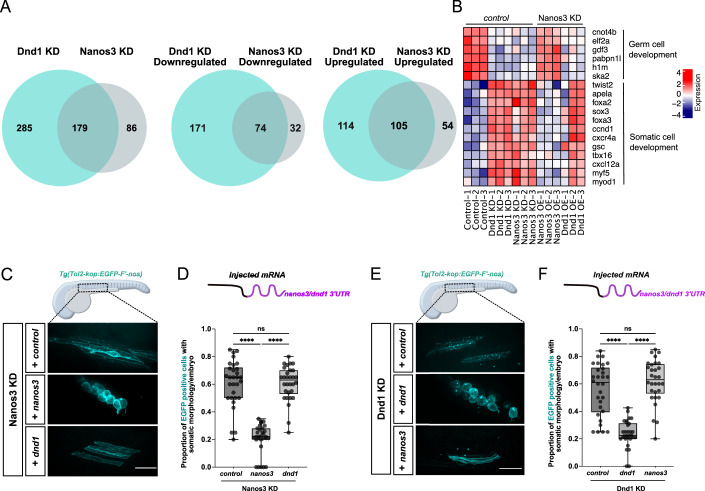
Nanos3 and Dnd1 regulate overlapping gene expression programs and function at the same step in PGC fate determination. (**A**) Venn diagrams showing the overlap of mRNAs whose levels are significantly altered in Nanos3 KD and Dnd1 KD PGCs relative to control PGCs based on bulk RNA sequencing. The left panel depicts the total number of differentially expressed transcripts in each condition, whereas the middle and right panels show the overlap of downregulated and upregulated transcripts, respectively. Differential expression was defined as adjusted *P* < 0.05. (**B**) Heatmap displaying the expression levels of transcripts related to somatic or germline development across various treatments as compared to the control. Nanos3 KD and Dnd1 KD PGCs show similar transcription profiles. Overexpression (OE) of *dnd1* RNA in Nanos3 KD embryos did not restore the transcriptome profile back to the control profile. Blue: downregulation; red: upregulation. (**C**, **D**) Representative images and quantification of somatic-like morphology of PGCs expressing membrane-bound EGFP. Nanos3 KD cells exhibit somatic morphology, which is restored to the characteristic round PGC morphology upon expression of morpholino-resistant *nanos3* RNA. *dnd1* RNA fails to restore PGC morphology in Nanos3 KD embryos. (**E**, **F**) Representative images and quantification of somatic-like morphology of PGCs expressing membrane-bound EGFP. Dnd1 KD cells exhibit somatic morphology, which is restored to the characteristic round PGC morphology upon expression of morpholino-resistant *dnd1* RNA. *nanos3* RNA fails to restore PGC morphology in Dnd1 KD embryos. (**C**–**F**) Data were obtained from *n* = 20 cells per condition from *N* = 3 independent experiments. Box plots show median (center line), interquartile range (box), and minimum to maximum values (whiskers). Individual data points are shown. Statistical significance was determined using the Mann–Whitney *U* test. ****P < 0.0001. Scale bar, 20 µm.

**Figure EV2:**
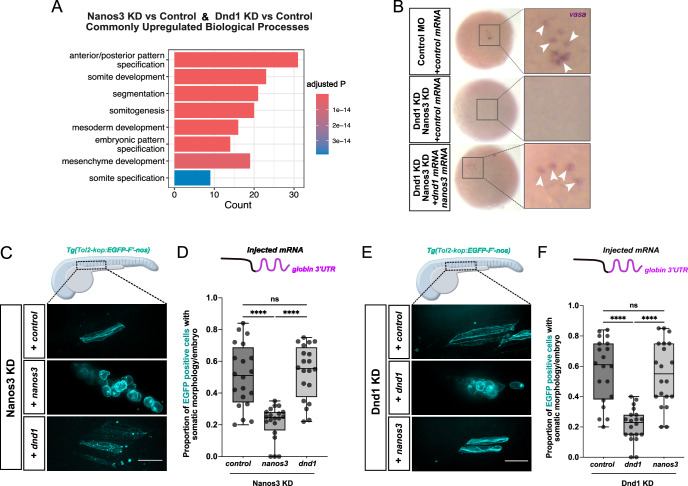
Additional analysis demonstrating the cooperative function of Nanos3 and Dnd1 in PGC maintenance. (**A**) Gene Ontology (GO) analysis showing biological processes enriched among transcripts upregulated both in Nanos3 KD and Dnd1 KD PGCs, compared to control. These processes include somatic differentiation and cellular reprogramming pathways. Bar length indicates the number of genes associated with each biological process. Statistical significance was assessed using a one-sided Fisher’s exact test. Adjusted *P* values are represented by the color scale. (**B**) Whole-mount in situ hybridization employing the PGC marker *vasa* on control embryos, embryos depleted of Dnd1 and Nanos3 co-injected with control RNA, and embryos depleted of Dnd1 and Nanos3 co-injected with morpholino-resistant *nanos3* and *dnd1* RNAs. A rescue is observed when both RNAs are injected (lower panel). White arrowheads point at PGCs. (**C**, **D**) Images showing cell morphology and quantification of PGCs labeled with membrane-bound EGFP. The cells display a somatic-like morphology in Nanos3 KD embryos, with a rescue to the characteristic round PGC morphology observed only when morpholino-resistant *nanos3* RNA carrying a *globin* 3′UTR was injected, but not with *dnd1* RNA carrying a *globin* 3′UTR. Scale bar: 20 µm. (**E**, **F**) Images demonstrating cell morphology and quantification of PGCs labeled with membrane-bound EGFP displaying somatic-like morphology in Dnd1 KD embryos, with a rescue to the characteristic round PGC morphology observed only when morpholino-resistant *dnd1* RNA carrying a *globin* 3′UTR was injected, but not with *nanos3* RNA carrying a *globin* 3′UTR. Scale bar: 20 µm. (**C**–**F**) Data were obtained from *n* = 20 cells per condition from *N* = 3 independent experiments. Box plots show median (center line), interquartile range (box), and minimum to maximum values (whiskers). Individual data points are shown. Statistical significance was determined using the Mann–Whitney *U* test. ****P < 0.0001. Scale bar, 20 µm.

To investigate the relationship between Nanos3 and Dnd1 further, we compared the RNA expression profiles of PGCs following the knockdown of each protein. Indeed, this analysis revealed a substantial overlap in differentially expressed RNAs between Nanos3- and Dnd1-depleted PGCs (Fig. 2A). Specifically, a heatmap of transcriptomic profiles revealed that PGCs lacking Nanos3 or Dnd1 exhibited a similar pattern, characterized by the downregulation of RNAs encoding for genes relevant to germ cell development and the upregulation of RNAs linked to somatic differentiation (Figs. 2B and EV2A). However, a subset of genes associated with somatic differentiation was affected in Dnd1 KD embryos but not in Nanos3 KD embryos and vice versa, suggesting that while the two proteins share many targets, each may also be involved in distinct molecular functions. Nevertheless, the strong similarity in RNA expression patterns is consistent with the idea that the primary function of Nanos3 and Dnd1 lies within a common regulatory framework that controls an RNA network essential for PGC identity.

Building on the similarity in RNA expression levels observed between the knockdown of either protein, we sought to determine whether Nanos3 and Dnd1 act within a linear regulatory hierarchy or function at the same step in maintaining PGC fate. Overexpression of *dnd1* in Nanos3 KD embryos did not restore PGC morphology, whereas expression of morpholino-resistant *nanos3* efficiently rescued the Nanos3 KD phenotype (Fig. 2C,D). Conversely, overexpression of *nanos3* failed to reverse the loss of PGC morphology induced by depletion of the Dnd1 protein, while the introduction of morpholino-resistant *dnd1* restored germ cell fate (Fig. 2E,F). As Dnd1 is known to regulate the stability and function of *nanos3* RNA via its 3’UTR (Kedde et al, 2007), we further employed Dnd1- and Nanos3-encoding RNA molecules that included the *globin* 3’UTR, which is not subjected to PGC-specific regulation (Köprunner et al, 2001). Similar to the results obtained with their own 3’UTRs, the *globin* 3’UTR-containing RNAs failed to reverse the respective cell morphology phenotype (Fig. EV2C–F). Co-injection of morpholino-resistant *nanos3* and *dnd1* RNAs with their own 3’UTRs into embryos knocked down for both proteins restored the expression of the germ cell RNA marker *vasa* to levels comparable to those in control PGCs (Fig. EV2B). Taken together, these findings show that neither protein can compensate for the loss of the other, consistent with the idea that Nanos3 and Dnd1 depend on each other’s functions to drive the same regulatory step required for maintaining germ cell fate.

### Nanos3 and Dead End1 form a complex in zebrafish PGCs

**Figure 3:**
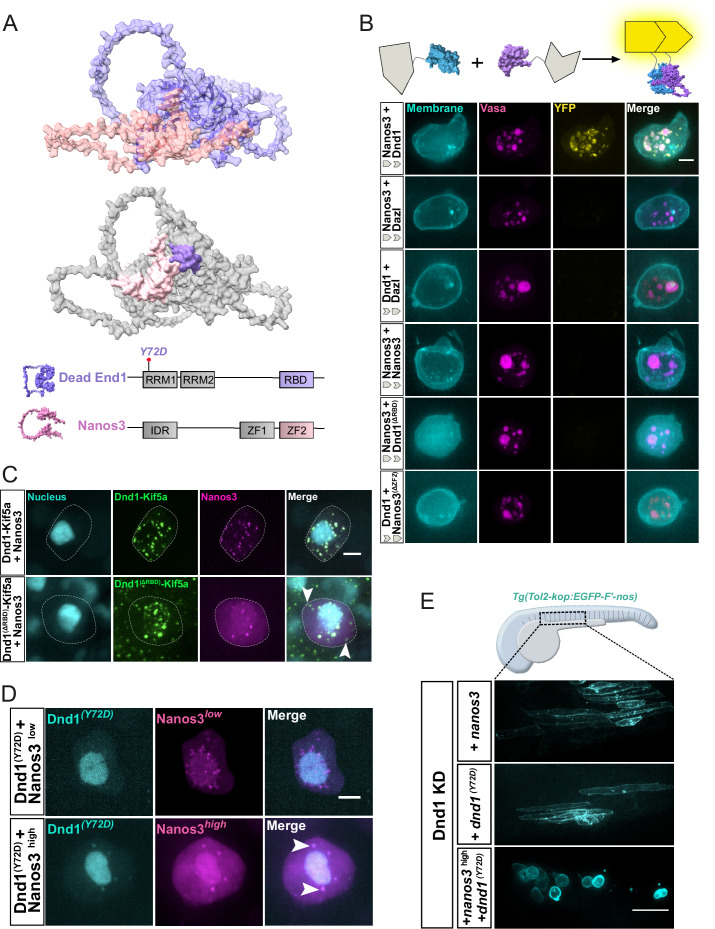
Nanos3 and Dnd1 form a complex mediated by the RBD of Dnd1 and the ZF2 domain of Nanos3. (**A**) AlphaFold2-based structure prediction showing the conformation of the zebrafish Nanos3 protein (pink) and Dnd1 protein (purple) complex (top). The interacting region within the zinc finger 2 (ZF2) domain of Nanos3 is highlighted in pink, and the interacting region within the RNA-binding domain (RBD) of Dnd1 is highlighted in purple. Domain schematics (bottom) indicate the position of the RNA recognition motif-1 (RRM1) of Dnd1, the position of the Y72D mutation (Tyrosine at 72^nd^ position was mutated to Aspartic acid), the RNA recognition motif-2 (RRM2), and the RNA-Binding Domain (RBD). The Nanos3 schematics show the intrinsically disordered region (IDR), Zinc finger-1 domain (ZF1), and Zinc finger-2 domain (ZF2). (**B**) Schematic illustrating the principle of the bimolecular fluorescence complementation (BiFC) assay, where a large fragment of YFP (gray) is fused to a protein of interest (POI), and a smaller fragment of YFP (gray) is fused to another POI. Upon interaction between the two proteins, the YFP is reconstituted and emits a yellow fluorescent signal. Symbols (gray) next to the proteins indicate fusion to a large or small YFP fragment. BiFC assay showing interaction between Nanos3 and Dnd1 in vivo. Fluorescent signal (YFP) indicates complex formation. *n* = 40 cells per condition, *N* = 3 experiments. Scale bar: 6 µm. (**C**) Kinesin-mediated relocalization assay. Kinesin family member 5 A (Kif5a) fused to Dnd1 protein (Green) affects the localization of wild-type Nanos3 protein (Magenta). This effect is inhibited by the deletion of the RNA-binding domain (RBD) of Dnd1. White arrowheads indicate Dnd1^ΔRBD^ fused to kinesin foci where Nanos3 protein is absent. Dotted lines signify the cell boundary. *n* = 25 cells per condition, *N* = 4 experiments. Scale bar: 6 µm. (**D**) Accumulation of the Dnd1^Y72D^ mutant protein in the nucleus (cyan) and its relocation upon strong overexpression of Nanos3 (magenta). White arrowheads in the bottom right panel point at the co-localization of the two proteins. *n* = 26 cells per condition, *N* = 3 experiments. Scale bar: 6 µm. (**E**) Representative images of PGC morphology in transgenic zebrafish embryos expressing membrane-bound EGFP in their germ cells. Expression of high levels of Nanos3 with the mutant Dnd1^Y72D^ maintains the characteristic round morphology of PGC in Dnd1 KD embryos. *n* = 30 cells per condition, *N* = 3 experiments. Scale bar: 20 µm.

**Figure EV3:**
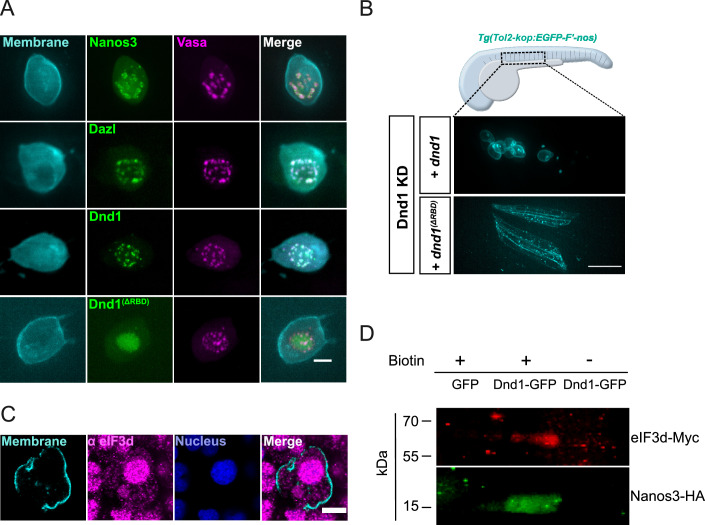
Localization and function of PGC-expressed proteins, and interactions among them. (**A**) Images showing wild-type localization of Nanos3, Dnd1, Dnd1^ΔRBD^, and Dazl GFP fusion proteins (green) relative to the cell membrane (cyan) and the germ cell granules (magenta). *n* = 32 cells per condition, *N* = 3 experiments. Scale bar: 6 µm. (**B**) Images showing the PGC morphology in transgenic zebrafish expressing membrane-bound EGFP at 24 h post fertilization. While Dnd1 can restore PGC round morphology and arrival at the gonad region in Dnd1 KD (upper panel), the Dnd1^ΔRBD^ form fails to do so. *n* = 36 cells per condition, *N* = 3 experiments. Scale bar: 20 µm. (**C**) Immunostaining of PGCs showing the endogenous eIF3d (magenta) localization. The merged image shows the presence of eIF3d in the nucleus and the cytoplasm. Scale bar: 2 µm. *n* = 8 cells, *N* = 3 experiments. (**D**) Proximity-dependent biotinylation assay. Transgenic (*Tg[bAct:mKate-p2A-TurboID-dGBP*]) embryos expressing cytoplasmic GFP or Dnd1–GFP were incubated in the presence or absence of biotin as indicated. Biotinylated proteins were isolated and analyzed by immunoblotting using anti-Myc (eIF3d–Myc, upper panel) and anti-HA (Nanos3–HA, lower panel) antibodies. Molecular weight markers (kDa) are indicated on the left.

Since Nanos3 and Dnd1 possibly act at the same level in regulating PGC fate, we considered the possibility that they form a protein complex. To explore this, we employed AlphaFold2 structural modeling (Jumper et al, 2021) to examine the potential associations between the two proteins. Indeed, this in silico analysis predicted an interaction mediated by the zinc finger 2 (ZF2) domain of Nanos3 and the RNA-binding domain (RBD) of Dnd1 (Fig. 3A; Movie EV1). To test this prediction experimentally, we conducted a bimolecular fluorescence complementation (BiFC) assay (Hu et al, 2002), in which two fragments of yellow fluorescent protein (YFP) are each fused to the proteins of interest, and fluorescence is emitted when the proteins physically interact to reconstitute a functional YFP protein. The resulting fluorescence signal revealed that the Nanos3–Dnd1 complex forms in the cytoplasm (Fig. 3B, top), with a distinct enrichment at the periphery of germ granules, a region where we previously observed PGC-specific RNA molecules co-localizing with ribosomes (Westerich et al, 2023b). The specificity of this assay was demonstrated by the reduction of the fluorescent signal when another germ granule-associated protein, namely Deleted in azoospermia-like (Dazl) (Maegawa et al, 1999) (Fig. EV3A), was placed instead of Nanos3 or Dnd1 (Fig. 3B). The specificity of the BiFC assay was further demonstrated by the lack of signal in the case of co-expression of Nanos3 protein fused to one fragment of the YFP and a Nanos3 protein fused to the complementary YFP fragment (Fig. 3B).

Consistent with the AlphaFold2 prediction that the RNA-binding domain (RBD) of Dnd1 interacts with the ZF2 domain of Nanos3, the deletion of either domain impaired BiFC signal intensity, indicating that the interaction between the two proteins is mediated through these specific regions (Fig. 3B). Interestingly, the Dnd1^ΔRBD^ protein is enriched in the nucleus at 10 h post fertilization (hpf) (Fig. EV3A). As assayed at 24 hpf, the Dnd1^ΔRBD^ protein was unable to restore germ cell fate in Dnd1-depleted embryos. Under these conditions, PGCs acquired somatic-like features (e.g., muscle-like morphology) (Fig. EV3B), suggesting that the interaction with Nanos3 is required for Dnd1 function in vivo. To further validate the Nanos3 and Dnd1 interaction, we fused Dnd1 to a kinesin motor domain (Kif5a), resulting in the relocalization of Dnd1 to discrete Kinesin-positive foci. While Nanos3 is normally found in the cytoplasm, nucleus, and germ granules (Fig. EV3A), co-expression with Kinesin-Dnd1 fusion led to enrichment of Nanos3 at the Kinesin foci, with a marked depletion from its native locations (Fig. 3C). Indeed, Kinesin-Dnd1^ΔRBD^ failed to direct Nanos3 to the ectopic locations further demonstrating the importance of the RBD for the interaction between the two proteins. Together, these findings support the conclusion that Nanos3 physically associates with Dnd1 in vivo.

Additional evidence for the interaction between the two proteins was obtained using the Dnd1^Y72D^ mutation in the RNA recognition motif-1 (RRM-1), which was previously shown to impair Dnd1 function and cause its aberrant accumulation in the nucleus at earlier stages of embryogenesis (Slanchev et al, 2009). Interestingly, overexpression of Nanos3 led to a significant portion of the Dnd1^Y72D^ protein exiting the nucleus, forming aggregates in the cytoplasm (Fig. 3D, arrowheads). Notably, under these conditions, the Nanos3–Dnd1^Y72D^ complex was able to support proper PGC development, indicating that the complex is sufficient to maintain germ cell fate (Fig. 3E). The BiFC analysis, kinesin-mediated relocalization, and Nanos3-dependent functional rescue of a Dnd1 mutant protein collectively establish the functional relevance of the Nanos3–Dnd1 complex, positioning it as a key molecular module that safeguards PGC identity.

### The Nanos3–Dead End1 complex interacts with the translation initiation machinery in vivo

**Figure 4:**
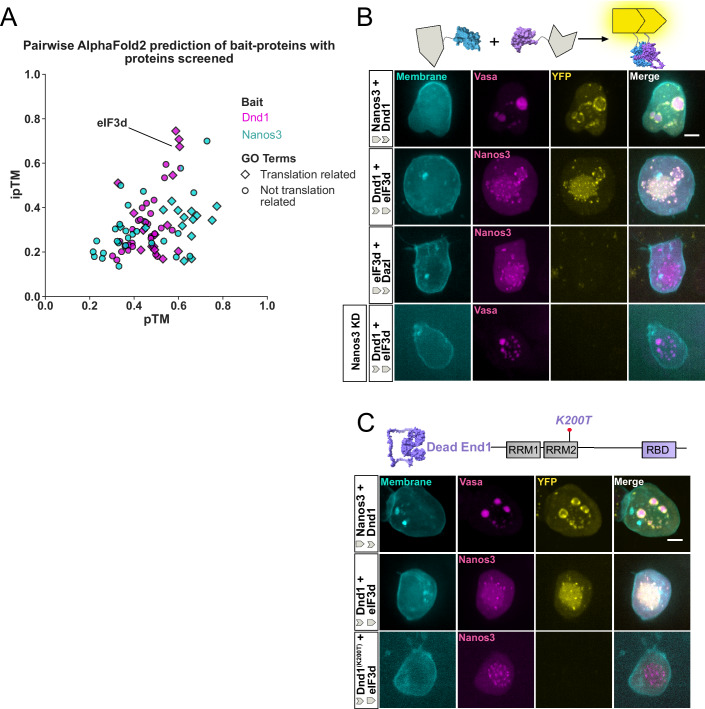
The Nanos3–Dnd1 complex interacts with translation initiation machinery proteins in zebrafish PGCs. (**A**) AlphaFold2-based interaction screen depicting predicted partners of zebrafish Nanos3 and Dnd1. Scatter plot of Predicted aligned error (pTM) versus interface-predicted TM score (ipTM) scores for each bait–prey pair; points are color-coded by bait (magenta, Dnd1; cyan, Nanos3) and shaped by Gene Ontology annotation (diamonds, translation-related; circles, non translation-related). eIF3d is a high-confidence interactor of Dnd1. (**B**) A scheme of the bimolecular fluorescence complementation (BiFC) assay (top), in which large and small fragments of YFP (gray) are fused to the indicated proteins of interest. Images showing Nanos3-dependent interaction of Dnd1 with eIF3d in vivo. Symbols (gray) next to the proteins indicate fusion to large or small YFP fragments. *n* = 30 cells per condition, *N* = 3 experiments. Scale bar: 6 µm. (**C**) A scheme of the domain organization of Dnd1 showing the RNA recognition motifs (RRM1, RRM2) and the RNA-binding domain (RBD), with the K200T (lysine residue at position 200 was mutated to threonine) mutation marked within the RRM2 domain. BiFC assay demonstrating that the wild-type Dnd1, but not the Dnd1^K200T^ version can interact with eIF3d. *n* = 32 cells per condition, *N* = 3 experiments. Scale bar: 6 µm.

Having shown that Nanos3 and Dnd1 physically associate with each other in zebrafish PGCs, we next examined whether this interaction extends to components involved in RNA translation regulation, proteins that are preferentially expressed in PGCs, as well as unrelated proteins (Dataset EV2). Structural predictions using AlphaFold2 revealed the highest predicted interaction scores of Nanos3 and Dnd1 with components of the translation initiation machinery. Notably, this analysis showed that eukaryotic translation initiation factor 3 subunit d (eIF3d) is predicted to associate with Dnd1 with a relatively high degree of confidence (Fig. 4A).

In zebrafish embryos, in situ hybridization analysis indicates that eIF3d is expressed uniformly (Thisse and Thisse, 2008). We further examined the subcellular localization of endogenous eIF3d in PGCs and detected the protein in both the cytoplasm and, interestingly, in the nucleus as well (Fig. EV3C). To experimentally validate the interactions predicted by the in silico screen, we performed a proximity-dependent biotinylation assay (Xiong et al, 2021) using Dnd1–GFP as bait. Under these conditions, both Nanos3–HA and eIF3d–Myc were detected, indicating that the Nanos3–Dnd1 complex resides in proximity to eIF3d (Fig. EV3D). To examine this point further and determine the location where the association among the proteins takes place, we employed the BiFC assay. This in vivo analysis showed a clear BiFC signal for eIF3d and Dnd1, which is co-localizing with Nanos3 (Fig. 4B, second row). To determine whether Nanos3 influences this association, we repeated the assay in Nanos3-depleted PGCs. The absence of a BiFC signal under these conditions suggests that stable interaction of Dnd1 with the translation initiation factor depends on the presence of Nanos3 (Fig. 4B).

Previous studies in *Xenopus* have shown that the RNA recognition motif-2 (RRM-2) of Dnd1 mediates the interaction with eIF3f (Aguero et al, 2017). To test whether this domain is also required for eIF3d–Dnd1 interaction in zebrafish, we employed Dnd^K200T^, a loss-of-function mutant in RRM-2, which was shown to be localized to the germ granules similar to the wild-type version (Slanchev et al, 2009). In this context, the BiFC signal was lost, indicating that the RRM-2 domain of Dnd1 is indeed important for the interaction with eIF3d (Fig. 4C). The evidence linking the Nanos3–Dnd1 complex to the translation initiation machinery suggests that Nanos3 functions in activating translation, which in turn can affect the localization of the RNA within germ granules.

### Nanos3 and Dead End1 control the translation and spatial distribution of RNAs encoding for germ plasm components

**Figure 5:**
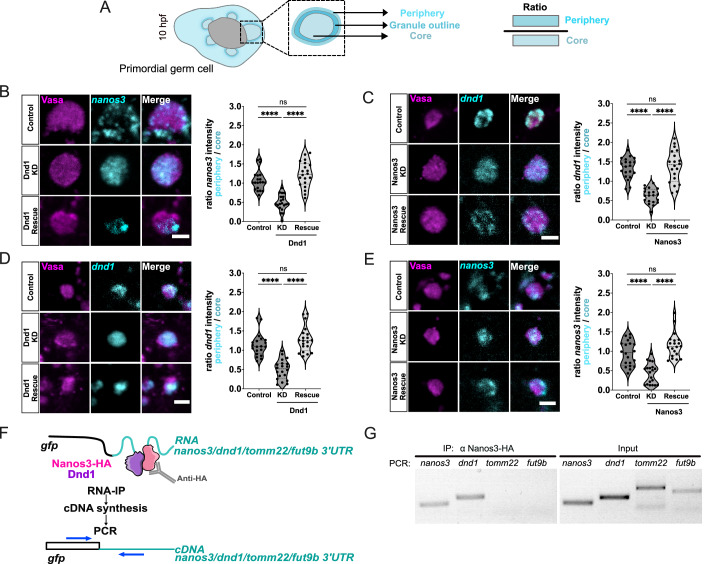
Nanos3 and Dnd1 control each other’s and their own RNA localization in germ granules. (**A**) Schematic illustration of a PGC with a magnified germ granule at 10 h post-fertilization (hpf). The inset presents the procedure used to calculate periphery-to-core intensity ratios to quantify the RNA distribution within and around the granule. (**B**–**E**) Images and quantification of endogenous RNA localization (cyan) across different conditions. Anti-Vasa antibody (magenta) marks germ granules. Quantifications show the periphery-to-core RNA intensity ratio. Data represent mean ± SD (*n* = 20 germ granules per condition, *N* = 3 experiments). Mann–Whitney *U* test; ****P < 0.0001; ns not significant. Scale bar: 2 µm. (**F**) Schematic representation of the RNA immunoprecipitation (RNA-IP) assay. One-cell stage embryos were injected with mRNAs encoding Nanos3-HA (pink) and Dnd1(purple) together with GFP reporter constructs carrying the 3′UTRs of *nanos3*, *dnd1*, *tomm22*, or *fut9b*. At 6 hpf, embryo lysates were subjected to immunoprecipitation using an anti-HA antibody (gray) to isolate Nanos3-associated RNA. Following RNA extraction and cDNA synthesis, PCR was performed to detect the bound transcripts. Magenta arrows indicate the forward and reverse primers used to amplify the respective regions of interest. (**G**) RNA-immunoprecipitation assay using antibodies directed against the HA-tagged Nanos3 followed by PCR analysis of co-precipitated 3’ UTRs of RNAs. *nanos3* and *dnd1* transcripts are enriched in the Nanos3-HA IP, whereas the negative controls, *tomm22* and *fut9b* show no signal. Input samples are shown on the right.

The findings presented above prompted us to investigate whether Nanos3 influences the translation and subcellular localization of RNAs encoding germ plasm components in zebrafish PGCs. Dnd1 has previously been shown to promote *nanos3* RNA translation and maintain the RNA at the periphery of the germ granules (Westerich et al, 2023b and Fig. 5A,B). To determine whether Nanos3 exerts a reciprocal regulatory role on *dnd1* RNA, we analyzed *dnd1* RNA distribution in Nanos3-depleted cells. Indeed, under these conditions, *dnd1* transcript levels were reduced at the periphery of the granule and concentrated at the core of the granule (Fig. 5C). A similar reduction in peripheral localization and a corresponding increase in RNA levels at the granule core were observed for *dnd1* RNA in cells knock down for Dnd1 protein (Fig. 5D), and for *nanos3* RNA in cells depleted of Nanos3 protein (Fig. 5E). To examine whether the Nanos3–Dnd1 complex physically associates with these RNAs, we performed RNA immunoprecipitation (RNA-IP) experiments. Indeed, using this assay, we could show that the 3’UTR of *nanos3* and *dnd1* transcripts interact with the Nanos3 protein (Fig. 5F,G). Together, these findings suggest that Nanos3 and Dnd1 jointly bind their own and each other’s RNAs, promote their association with translation machinery, and the localization of the RNAs to the granule periphery.

**Figure EV4:**
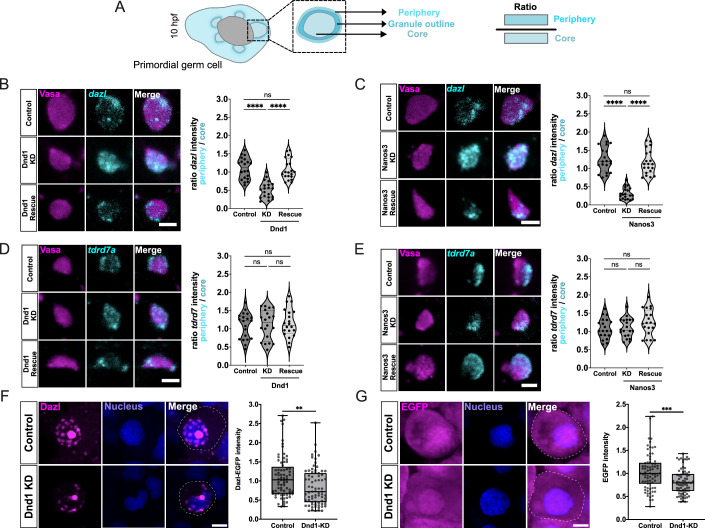
The role of Nanos3 and Dnd1 in controlling the localization of other RNAs and protein production. (**A**) Schematic illustration of a PGC with a magnified germ granule at 10 h post-fertilization (hpf). The inset presents the procedure used to calculate periphery-to-core intensity ratios to quantify the RNA distribution within and around the granule. (**B**–**E**) Images and quantification of different endogenous RNA localization (cyan) across the different experimental conditions. Anti-Vasa antibody (magenta) marks germ granules. Quantifications on the right of each panel present the periphery-to-core RNA intensity ratio. Scale bar: 2 µm. *n* = 20 cells per condition *N* = 3. Data represent mean ± SD. Mann–Whitney *U* test; ****P < 0.0001; ns: not significant. (**F**) Images and quantification of Dazl protein levels in control and Dnd1 KD embryos, injected using the *dazl–EGFP–dazl 3*′*UTR* reporter RNA. Dotted lines indicate cell boundaries. Scale bar: 5 µm. Quantification was performed from *n* = 75 cells per condition obtained from *N* = 3 independent experiments. Box plots show median (center line), interquartile range (box), and minimum to maximum values (whiskers). Individual data points are shown. Statistical significance was determined using the Mann–Whitney *U* test. ****P < 0.0001. (**G**) Images and quantification of EGFP protein levels in control and Dnd1 KD embryos, injected using the *EGFP–globin 3*′*UTR* reporter RNA. Dotted lines indicate cell boundaries. Scale bar, 5 µm. Quantification was performed from *n* = 75 cells per condition obtained from *N* = 2 independent experiments. Box plots show median (center line), interquartile range (box), and minimum to maximum values (whiskers). Individual data points are shown. Statistical significance was determined using the Mann–Whitney *U* test. ****P < 0.0001.

Importantly, similar to the dependence of *nanos3* and *dnd1* RNAs localization on the proteins they encode, we found that the positioning of *dazl* RNA also relies on both Nanos3 and Dnd1 proteins (Fig. EV4A–C). This suggests the regulatory function described here extends to additional germ cell RNAs. In contrast, the localization of Tudor domain-containing protein 7 (*tdrd7a*) RNA remained unaffected by either Nanos3 or Dnd1 depletion, indicating that the regulation of certain RNA molecules’ function is not dependent on the Nanos3–Dnd1 complex (Fig. EV4D,E; Westerich et al, 2023b).

**Figure 6:**
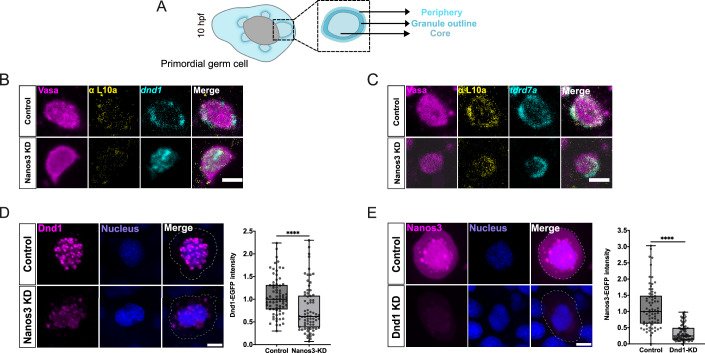
Nanos3 and Dnd1 control RNA positioning and protein expression in zebrafish PGCs. (**A**) Schematic illustration of a PGC with a magnified germ granule at 10 h post-fertilization (hpf). (**B**, **C**) Localization of endogenous RNAs (*dnd1* and *tdrd7a*, cyan) relative to the ribosomal marker L10a (yellow) is shown in control and Nanos3 KD PGCs. Scale bar: 2 μm. *n* = 20 cells per condition, *N *= 2 experiments. (**D**) Images and quantification of Dnd1 protein levels in control and Nanos3 KD embryos, injected with the *dnd1–mGFP–dnd1 3*′*UTR* reporter RNA. Dotted lines indicate cell boundaries. Scale bar: 5 µm. Quantification was performed from *n* = 75 cells per condition obtained in *N* = 3 independent experiments. Box plots show median (center line), interquartile range (box), and minimum to maximum values (whiskers). Individual data points are shown. Statistical significance was determined using the Mann–Whitney *U* test. ****P < 0.0001. (**E**) Images and quantification of Nanos3 protein levels in control and Dnd1 KD embryos, assessed using the *EGFP–nanos3–nanos3 3*′*UTR* reporter RNA. Dotted lines indicate cell boundaries. Scale bar: 5 µm. Quantification was performed from *n* = 75 cells per condition obtained in *N* = 3 independent experiments. Box plots show median (center line), interquartile range (box), and minimum to maximum values (whiskers). Individual data points are shown. Statistical significance was determined using the Mann–Whitney *U* test. ****P < 0.0001.

These findings prompted us to examine whether depleting Nanos3 or Dnd1 actually affects their own RNA translation. To this end, we first examined the localization of L10a, a component of the 60S ribosomal subunit that serves as a marker of the translational machinery. Under all conditions tested, L10a remained at the periphery of germ granules (Fig. 6B,C). However, in Nanos3-depleted embryos, *dnd1* RNA but not *tdrd7a* RNA accumulated in the granule core, spatially separated from L10a (Fig. 6B,C). This relocalization can reduce the translational efficiency as the RNA is displaced from zones where the translation machinery is more abundant.

Consistent with the results presented above, Knockdown of Nanos3 led to a pronounced reduction in Dnd1 protein levels (~30%) (Fig. 6D) and similarly, Nanos3 protein levels were strongly decreased in Dnd1 KD embryos (~75%) (Fig. 6E). In addition, we observed decreased Dazl protein levels following Dnd1 depletion (~30%) (Fig. EV4F), suggesting that the Nanos3–Dnd1 complex supports the translation of other germline determinants. Whereas the *globin 3’UTR*-linked GFP reporter showed a significant but comparatively small decrease upon depletion of Dnd1 (~20%) (Fig. EV4G), suggesting indirect effects on the translation of other RNAs. Together, these findings show that Nanos3 and Dnd1 are critical for interaction with the translation machinery, thereby promoting the translation of key germline proteins and maintaining the localization of their RNAs at the granule periphery.

## Discussion

**Figure 7:**
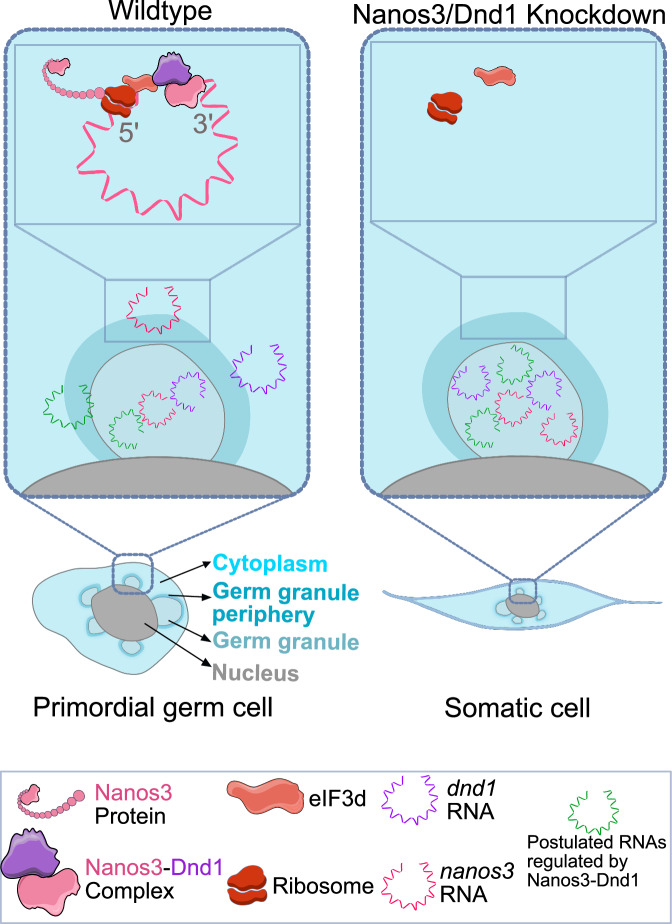
Proposed model illustrating the role of the Nanos3–Dnd1 complex in regulating PGC identity. The Nanos3–Dnd1 complex promotes germline mRNAs translation at the germ granule periphery. In wild-type PGCs (left), Nanos3–Dnd1 binds the 3′ UTR of *nanos3* and interacts with the cap binding initiation factor eIF3d, promoting the formation of a closed-loop mRNA configuration. The Nanos3–Dnd1 complex can bind its corresponding RNAs as well as postulated targets and promote their translation, thereby contributing to the maintenance of PGC fate during early embryogenesis. In the absence of Nanos3 or Dnd1 (right), these RNAs are not associated with the ribosomes and translocate into the germ granule core, leading to reduced protein synthesis and somatic reprogramming of the germline.

In this study, we show that germ cell fate in zebrafish is maintained by a protein complex containing Nanos3 and Dnd1. Consistent with this, Dnd1 and Nanos3 show similar loss-of-function cellular phenotypes, regulate largely overlapping sets of mRNAs, and each depends on the other for translation and localization of its own RNA. Nanos3 and Dnd1 bind the 3′ UTRs of their target RNAs (Sonoda and Wharton, 1999; Kedde et al, 2007; preprint: Suzawa et al, 2025; Fig. 5F), whereas eIF3d interacts with the 5′ cap of the RNAs (Lee et al, 2016). Together with our protein interaction data, these observations support a model in which upregulation of RNA translation by Nanos3 and Dnd1 involves communication between the two RNA ends, in line with the “closed loop” model of mRNA translation (Tomek and Wollenhaupt, 2012; Rissland, 2017; Vicens et al, 2018; Lai et al, 2018; Ermolenko and Mathews, 2021) (Fig. 7). We have previously shown that, in zebrafish PGCs, RNAs exhibit very rapid mobility within germ granules that contain translation-repression factors, whereas translation-associated components and RNA translation are observed outside these condensates (Westerich et al, 2020, 2023b). By contrast, recent work in *Drosophila* reports the 5′ and 3′ ends of *nanos* RNA to be spatially separated, with the 5′ end pointing outwards and the 3′ UTR anchored at the granule center, in which ribosomes are abundant (Chen et al, 2024; Ramat et al, 2024). It will therefore be interesting to determine whether germ granules in other invertebrates and vertebrates adopt a *Drosophila*-like organization, with anchored 3′ UTRs that are depleted from the region where translation is proposed to be activated.

More broadly, RNA localization and translational control are central to germ cell development in several organisms (Ephrussi et al, 1991; Lipshitz and Smibert, 2000; Rangan et al, 2009; Parker et al, 2020). Germline RNAs are often translated through alternative initiation pathways shaped by specific RNA–protein assemblies (Sadato et al, 2018; Aguero et al, 2017; Huggins and Keiper, 2020), which in our case include the eIF3d subunit. It will be important to determine whether eIF3d-containing complexes play comparable roles in germ cells of other species and at different stages of germline development. While our results are consistent with a direct role for the Nanos3–Dnd1–eIF3d complex in activating translation, this point could be further supported by in vitro experiments in which the function of the complex can be tested in isolation.

The role of Nanos3 described here differs from the canonical function of Nanos, which has been shown to function as a translational repressor acting with Pumilio to inhibit Hunchback protein production (Irish et al, 1989; Sonoda and Wharton, 1999). Nanos3 is the only known isoform to be expressed in zebrafish PGCs. During early embryogenesis, we find Nanos3 in complex with Dnd1, where it contributes to translational activation. Dnd1 has been reported to promote protein production by enhancing RNA stability and translation through antagonizing miRNA-mediated repression (Kedde et al, 2007), Our findings suggest that, when in complex with Dnd1, in addition to the translation initiation activation, Nanos3 could function in antagonizing miRNA function as well. Similar context-dependent function has been described for other RNA-binding proteins, including PUF family members, whose activity is shaped by cellular context, RNA targets, and binding partners (Quenault et al, 2011). Together, these observations support a view of Nanos3 as a regulator that can function as either a repressor or an activator depending on its associated cofactors (e.g., CNOT complex (Suzuki et al, 2014) or Dnd1, respectively). While our data identify Dnd1 as a key determinant of this activity in vivo, the involvement of additional intermediate components cannot be excluded, and resolving the underlying mechanisms will require complementary in vitro biochemical approaches in other systems.

The formation of different Nanos3 or Dnd1-containing complexes may also contribute to the transcriptome differences observed under each knockdown condition. In addition to controlling their own RNAs and that of *dazl*, the Nanos3–Dnd1 complex may influence other RNAs expressed preferentially in the germline, including *h1m* (Müller et al, 2002; Wibrand and Olsen, 2002), *sod2* (Tarbashevich et al, 2023), *vasa* (Castrillon et al, 2000), *gra* (Strasser et al, 2008) and *hsp90* (Pfeiffer et al, 2018), as well as RNAs that are also expressed in other cell types. Whether these RNAs are directly bound by the Nanos3–Dnd1 complex or are affected indirectly remains to be determined.

Beyond their physiological role in germ cell development, both Dnd1 and Nanos3 have been linked to pathological conditions, including germ cell-derived tumors and infertility (Kusz-Zamelczyk et al, 2013; Imai et al, 2020; Westerich et al, 2023a). Studying the role of the Nanos3–Dnd1–eIF3d complex in these contexts will be an important approach to define the signaling pathways controlled by Nanos proteins and Dnd1, and towards understanding how their misregulation contributes to disease.

## Methods

**Table:** Reagents and tools table

Reagent/resource	Reference or source	Identifier or catalog number
**Experimental models**		
Zebrafish: wild-type strain of the AB(TL) background	N/A	N/A
Zebrafish: *tg*(*kop*:*mcherry-f’-nos3’UTR-cmlc*:*egfp*)	Tarbashevich et al, 2015	N/A
Zebrafish: *tg*(*-1*.*8unc45b*:*tfp*)	Etard et al, 2015	ZFIN: ZDB-TGCONSTRCT-160322-1
Zebrafish: *tg(kop-egfp-f-nos-3’ UTR;cry:dsred)*	Blaser et al, 2005	ZFIN: ZDB-ALT-070406-1
Zebrafish: *tg[bAct:mKate-p2A-TurboID-dGBP]*	Xiong et al, 2021	ZFIN: ZDB-TGCONSTRCT-220228-2
**Deposited data**		
Bulk RNA Sequencing	This study	PRJNA1357867
**Antibodies**		
Rabbit polyclonal anti-Vasa	GeneTex	Cat# GTX128306, RRID: AB_2847856
Chicken polyclonal anti-GFP-1020	Aves Labs	Cat# GFP-1010, RRID: AB_2307313
Anti-mouse Alexa488	Thermo Fisher	Cat# A-11029, RRID: AB_2534088
Anti-mouse Alexa568	Thermo Fisher	Cat# A-11031, RRID: AB_144696
Anti-rabbit Alexa488	Thermo Fisher	Cat# A-11034, RRID: AB_2576217
Anti-rabbit eIF3d Polyclonal Antibody	Thermo Fisher	#PA5-23293, RRID: AB_2540817
Anti-mouse RPL10a Monoclonal Antibody	Sigma-Aldrich	Cat# WH0004736M1, RRID: AB_1843413
Anti-mouse Myc Monoclonal Antibody	Sigma-Aldrich	Cat# MABE282, RRID: AB_11213164
Anti-Rat HA Monoclonal Antibody	Sigma-Aldrich	Cat# 11867423001, RRID: AB_390918
Goat Anti-Mouse IgG 680RD	Licorbio	Cat# 926-68070, RRID: AB_10956588
Goat Anti-Rat IgG 680RD	Licorbio	Cat# 926-68076, RRID: AB_10956590
**Oligonucleotides and other sequence-based reagents**		
Injected RNAs	See Table EV1	See Table EV1
Morpholinos	See Table EV1	See Table EV1
RNAscope detection probes	See Table EV1	See Table EV1
**Chemicals, enzymes, and other reagents**		
Tricaine	Sigma	Cat# A5040
HEPES	Fisher BioReagents	Cat# BP299-500
PFA	Sigma	Cat# 158127
BSA	Sigma	Cat# A8022
Sheep serum	Sigma	Cat# S3772
Triton X 100	Carl Roth	Cat# 3051.2
Tween-20	AppliChem	Cat# A4974
Proteinase K	Roche	Cat# 0311583600
DAPI	ACD	Cat# 320858
DMSO	AppliChem	Cat# A3672
**Critical commercial assays**		
Manual RNAscope Multiplex Fluorescent Detection Reagents	ACD	Cat# 320851
RNAscope Protease III and Protease IV reagents	ACD	Cat# 322340
mMessage mMachine sp6 Kit	Thermo Fisher	Cat# AM1340
mMessage mMachine T3 Kit	Thermo Fisher	Cat# AM1348
mMessage mMachine T7 Kit	Thermo Fisher	Cat# AM1344
**Software**		
Fiji (version 2.0.0-rc-43/1.51a)	NIH	https://fiji.sc/
Huygens Professional (version 19.04)	SVI	http://svi.nl
Prism (version 9.4.0)	Graphpad	https://www.graphpad.com/scientific-software/prism/
VisiView (version 4.0.0.14)	Visitron Systems GmbH	http://www.visitron.de/Products/Software/VisiView/visiview.html
ZEN (version 2010B SP1, 6.0)	Zeiss	https://www.zeiss.com/microscopy/en/products/software/zeiss-zen.html

### Key resources table

Reagents and tools table is individually provided.

### Experimental models and subject details

#### Zebrafish husbandry and embryo collection

Wild-type *Danio rerio* (AB or AB/TL genetic background) were maintained at 28.5 °C under standard facility conditions. Embryos were obtained through natural mating and staged by hours post-fertilization (hpf) (Kimmel et al, 1995); several transgenic lines were utilized for in vivo analyses. The *Tg (kop: egfp-f’-nos3*′*UTR-cry: dsred)* line expresses membrane-targeted EGFP in PGCs, driven by the maternal *askopos* (*kop)* promoter and regulated post-transcriptionally via the *nanos3* 3′ untranslated region (3′UTR), ensuring RNA localization to germ plasm and PGC-specific translation (Köprunner et al, 2001; Blaser et al, 2005). Similarly, the *Tg (kop: mcherry-f’-nos3*′*UTR-cmcl: egfp)* line directs mCherry to PGC membranes under the same regulatory elements (Tarbashevich et al, 2015). To label somatic tissues, particularly developing musculature, the *Tg (-1.8unc45b: TFP)* line was employed, which expresses TFP under the control of a muscle-specific promoter (Etard et al, 2015). Embryos were collected in 0.3X Danieau’s buffer (17.4 mM NaCl, 0.21 mM KCl, 0.12 mM MgSO₄·7H₂O, 0.18 mM, Ca(NO₃)₂, 1.5 mM HEPES, pH 7.6) at designated incubation temperatures of 25 °C, 28 °C, or 31 °C, depending on experimental requirements. All the experiments and fish maintenance were performed following the regulations of the North Rhine-Westphalian Office of Nature, Environment and Consumer Protection (LANUV NRW).

#### Cloning of DNA constructs

For cloning plasmid constructs, a restriction-free cloning approach (Van Den Ent and Löwe, 2006) was employed. Mutations within the sequence were induced via PCR-based site-directed mutagenesis technique adapted from the QuickChange^TM^ site-directed mutagenesis protocol (Zheng et al, 2004) with overlapping primers. The constructs with either N- or C- terminal fragments of the YFP were cloned by replacing the GFP sequence of the template plasmid harboring the protein of interest (POI), namely GFP-POI-nos 3′UTR. All BiFC experiments were performed using the minimal RNA concentration required to clearly visualize primordial germ cells. A list of mRNAs used in this study is provided in the Reagents and Tools table.

#### Microinjections

Capped synthetic mRNAs and morpholino oligonucleotides (MOs) were prepared using the mMESSAGE mMACHINE kit (Thermo Fisher Scientific) in accordance with the manufacturer’s protocol. Morpholinos designed to inhibit translation were sourced from Gene Tools, LLC (Philomath, Oregon). Microinjections were performed at the one-cell stage, delivering 2 nL of the RNA and/or morpholino solution into the cell using a glass capillary needle mounted on a PV830 Pneumatic PicoPump injector (World Precision Instruments). All injection series included both experimental and matched control groups and were processed in parallel under identical handling conditions. Detailed concentrations of injected reagents are summarized in Table EV1.

#### Image acquisition and processing

Embryos were dechorionated prior to image acquisition, transferred to 1.5% agarose ramps covered with 0.3× Danieau’s solution, and maintained at 28 °C during imaging. Embryos at 1 dpf were anesthetized using 0.64 mM Tricaine (Sigma-Aldrich) in 0.3X Danieau’s solution prior to imaging. For Figs. 1, 5, and 6, confocal images were acquired on a Zeiss LSM 710 microscope equipped with 405 nm, 488 nm, 561 nm, and 633 nm lasers and a 63X W Plan-Apochromat objective (Zeiss). Image acquisition was controlled using ZEN software (Zeiss, version 2010B SP1, 6.0). For Figs. 2, 3, and 4, spinning disk confocal microscopy was performed using Carl Zeiss Axio Imager Z1 and M1 microscopes outfitted with Yokogawa CSUX1FW-06P-01 spinning disk modules. Fluorescence images were captured with a Hamamatsu ORCA Flash 4.0 LT C11440 camera, and data collection was managed using VisiView software (Visitron, version 4.0.0.14). All image processing and quantitative analyses were performed using Fiji (ImageJ) (NIH, version 2.0.0-rc-43/1.51 d 2015). All experiments were performed using independent biological replicates and, where applicable, repeated technical replicates to ensure reproducibility.

#### Analysis of a muscle marker expression in germ cells

To assess aberrant expression of a muscle-specific marker in germ cells lacking endogenous Nanos3 protein, fluorescence from TFP expressed under the control of the *unc45b* promoter was quantified in both PGCs and neighboring muscle precursor cells. This analysis was conducted as described previously (Gross-Thebing et al, 2017). Using Fiji software (NIH, version 2.0.0-rc-43/1.51 d 2015), a region of interest was manually defined around the boundary of each germ cell and applied to the channel displaying the muscle marker signal. The mean fluorescence intensity within the germ cell was recorded. The same region of interest was then repositioned over two adjacent muscle cells, and mean fluorescence intensities were measured accordingly. The ratio of the mean signal intensity between the PGCs and surrounding muscle tissue was calculated to determine relative marker expression.

#### Bulk RNA sequencing

Embryos were dechorionated at 9–10 hpf using pronase and rinsed three times in egg water supplemented with methylene blue. To isolate single cells, embryos were dissociated in Ca^+^/Mg2^+^-free dissociation buffer (Gibco) at a 2:1 buffer-to-embryo volume ratio. The resulting cell suspension was filtered through a 40-μm nylon mesh (Falcon) into ice-cold 1× phosphate-buffered saline (PBS) and maintained on ice prior to fluorescence-activated cell sorting (FACS). Sorting was carried out on a FACSAria Illu system (BD Biosciences) equipped with a 85-μm nozzle, using GFP fluorescence to identify and isolate PGCs. Sorted cells were collected into 100 μL 1× PBS, immediately mixed with a threefold excess of TRIzol reagent (Invitrogen), and snap-frozen in liquid nitrogen for downstream RNA isolation.

RNA extraction and sequencing were performed by Single Cell Discoveries (Utrecht, Netherlands), and data processing was carried out in R version 4.2.1 (R Core Team, 2021). Differential gene expression was assessed using the DESeq2 package (Love et al, 2014), with genes exhibiting a *P*-adjusted value < 0.05 considered differentially expressed. Volcano plots were generated using the Enhanced Volcano package (Blighe et al, 2021). Gene Ontology (GO) enrichment analysis of biological processes was conducted using the Cluster Profiler package (Wu et al, 2021).

#### Evaluation of somatic cell shape acquisition by germ cells

To establish a defined criterion for somatic cell morphology, germ cells were visually compared to three representative somatic cell types, namely muscle, notochord, and neuronal cells. A germ cell was classified as having adopted a somatic morphology only if its shape closely resembled that of one of these reference cell types. Subsequently, the ratio between the number of germ cells with somatic cell shape and that with the wild-type germ cell shape was calculated. It is important to note that the proportion of germ cells displaying transdifferentiation under knockdown conditions may thus be underestimated. Subtle morphological changes or transitions toward somatic identity that fall outside the predefined categories were not included in the analysis and, therefore, may not be reflected in the reported analysis.

#### In situ hybridization

Chromogenic in situ hybridization was performed as described previously (Thisse and Thisse, 2008). Briefly, embryos at 7 hpf were fixed in 4% paraformaldehyde (PFA) in 1× PBS overnight at 4 °C, then dechorionated. These samples were then stored in 100% MeOH at -20 °C. Embryos were permeabilized using 10 mg/ml Proteinase K (Roche) in PBT (0.1% Tween in 1× PBS) for 30 s and fixed again for 1 h. Hybridization was performed using digoxigenin-labeled probes that were synthesized according to the manufacturer’s protocol (Roche). Detection of the probes was performed by incubating the embryos with an anti-digoxigenin antibody coupled to alkaline phosphatase (Roche) diluted 1:5000 in blocking solution (0.1% Tween in 1× PBS, 2% sheep serum, 2 mg/ml BSA). The staining reaction was performed using NBT/BCIP (Roche) in NTMT buffer (100 mM Tris HCl pH 9.5, 50 mM MgCl2, 100 mM NaCl, 0.1% Tween). PGCs were counted on a Zeiss SteREO Discovery V12 binocular and processed using Fiji software (NIH, version 2.0.0-rc-43/1.51 d 2015).

#### AlphaFold2 multimer analysis

Protein sequences were sourced from the UniProt database. For predicting protein-protein interactions, AlphaFold2 (version 2.3) was employed to conduct pairwise multimer predictions between bait and candidate proteins (preprint: Evans et al, 2021). The predicted template modeling score (pTM) and interface-predicted template modeling score (ipTM) were extracted from the highest-scoring relaxed prediction. The baits (Dead End1 [UniProt: Q7T1H5] and Nanos3 [UniProt: Q90WW1]) were predicted with a list of selected candidates associated with germ plasm and translation initiation. The Gene Ontology (GO) terms considered were “translation” (GO:0006412) alongside its child terms “translational initiation” (GO:0006413) and “translation initiation factor activity” (GO:0003743). The code is available on GitHub (https://github.com/Julian-We/PPInet).

#### RNA immunoprecipitation

One-cell stage wild-type embryos were injected with a mixture of six mRNAs: *nanos3-HA-globin 3’UTR, dnd1-FLAG-globin 3’UTR, GFP-nanos3 3’UTR, GFP-dnd1 3’UTR, GFP-tomm22 3’UTR,* and *GFP-fut9b 3’UTR*. At 5-6 hpf, embryos were dechorionated manually and dissociated into single cells using enzyme-free Cell Dissociation Buffer (Gibco). Cells were then undergone centrifugation at 1000 rpm, washed twice with 500 µl Cell Dissociation Buffer, and snap-frozen in liquid nitrogen. Cell pellets were lysed in 500 µl IP Lysis Buffer (Pierce) supplemented with Protease inhibitor cocktail (Thermo Fisher Scientific), RiboLock RNAse inhibitor (Thermo Fisher Scientific), and 500 µm dithiothreitol (DTT). Lysates were cleared by centrifugation at 16,000 rpm for 15 min, 4 °C. In parallel, 30 µl Dynabeads Protein G (Thermo Fisher Scientific) solution per sample was washed twice with 500 µl IP Lysis Buffer (Pierce) and coated for 1 h with 2 µg of either mouse anti-HA antibody (Sigma-Aldrich, H9658) or with rabbit pan-actin antibody (Cell Signalling Technology, 4968) in a total volume of 500 µl IP Lysis Buffer (Pierce) supplied with Protease inhibitor cocktail (Thermo Fisher Scientific) at room temperature. Unbound antibody was removed by washing beads twice with 500 µl IP Lysis Buffer (Pierce). Clarified cell lysates were incubated with antibody-coated beads for 2 h with gentle mixing at 4 °C. Input samples (50 µl of each cell lysate) were retained for comparative analysis. Following incubation, beads bound to the Ribonucleoprotein (RNP) complexes were washed twice with 500 µl IP Lysis Buffer (Pierce), supplied with Protease inhibitor cocktail (Thermo Fisher Scientific), RiboLock (Thermo Fisher Scientific), and 500 µm dithiothreitol (DTT). RNP complex was eluted by incubating the beads in 1 ml of TRIzol (Thermo Fisher Scientific) for 10 min at 70 °C. RNA was isolated using the standard TRIzol-based RNA-extraction protocol. RNA pellets were resuspended in 25.4 µl nuclease-free water and reverse transcribed into cDNA using the OmniScript RT Kit (Qiagen) according to the manufacturer’s instructions with 40 µl final volume of each reaction. Reaction mixes were incubated at 37 °C for 1 h, and cDNAs samples were stored at -20 °C. To visualize bound mRNAs, PCR was performed using primers for targets of interest (*nanos3* and *dnd1*; *tomm22* and *fut9b* served as negative controls). Amplification was performed using 2X Phusion (Thermo Fisher Scientific) mix reactions according to the manufacturer’s instructions. Results presented in the manuscript were obtained with 58 °C annealing temperature and 20 s elongation steps in 30 cycles.

#### RNA and protein detection in whole-mount embryos

For all experimental conditions, embryos were fixed overnight at 4 °C in 4% PFA prepared in 1× PBS, with gentle agitation. To enable simultaneous detection of mRNA and protein within PGCs, a multiplexed in situ hybridization approach was performed using the RNAscope Fluorescent Multiplex Reagent Kit (ACD Bio), as previously described (Gross-Thebing et al, 2014) with probe sets specific for *nanos3*, *dnd*1, *tdrd7a*, and *dazl*. Following hybridization, embryos were processed for immunofluorescence using a modified protocol. Primary antibodies included rabbit anti-Vasa (GeneTex, GTX128306; 1:400 dilution), rabbit anti-eIF3d (Thermo Fisher, #PA5-23293; 1:100 dilution), and chicken anti-GFP (Aves Labs, GFP-1020; 1:600 dilution). Fluorescently labeled secondary antibodies were applied as follows: Alexa Fluor 488-conjugated anti-chicken IgY (Jackson ImmunoResearch; 1:600) for GFP and Alexa Fluor 568-conjugated anti-rabbit IgG (Thermo Fisher Scientific, A-11031; 1:1000) for Vasa and eIF3d. Embryos were mounted on agarose-coated ramps with 1× PBS for imaging.

#### Measurement of the periphery–core ratio of RNA within germ granules

To quantify the spatial distribution of RNA within germ granules, as depicted in Fig. 5, the relative enrichment of signal at the granule periphery versus the core was measured. Image segmentation and analysis were performed using Fiji software (NIH, version 2.0.0-rc-43/1.51 d 2015). A binary mask was generated from the Vasa signal to define granule boundaries, and morphological operations were applied to delineate distinct regions corresponding to the core and peripheral zones in accordance with (Westerich et al, 2023b). Mean fluorescence intensities were then extracted from each region, and the ratio of peripheral-to-core RNA signal was calculated to assess sub-granular localization.

#### Quantifications of protein levels

Images were deconvolved using Huygens Professional. Deconvolution settings included automatic background subtraction, signal-to-noise ratios (SNRs) of 2 for the EGFP-Nanos3, EGFP-Dazl, EGFP, H2A-tagBFP, and mScarlet-i-NLS channels. For the Dnd1-mGFP channel, the signal-to-noise ratios (SNRs) were 1.5. Images were deconvolved using a theoretical point spread function (PSF) and 25 maximal iterations with a quality change threshold of 0.05%. Fluorescence intensities of the GFP and BFP channels within whole cells were measured in Fiji on “Sum Slices” Z-projections. Cell outlines were generated manually based on the signal in the nucleus channel. Intensity ratios were calculated by dividing the GFP intensity by the BFP intensity, as the nucleus expression is not affected by the knockdown of either Nanos3 or Dnd1. To allow for better comparisons, the values were then normalized by the median of ratios within the control group.

#### Proximity-dependent biotin labeling assay

Transgenic zebrafish *Tg[bAct:mKate-p2A-TurboID-dGBP]* embryos (Xiong et al, 2021) at 1-cell stage were injected with *nanos3-HA-globin3’UTR* mRNA. For the control sample (sample 1), the cytoplasmic GFP was employed as a target protein for the TurboID together with *dnd1-globin3’UTR* mRNA. In sample 2, Dnd1-GFP (*dnd1-gfp-globin3’UTR*) was expressed instead of the untagged Dnd1. Both samples were co-injected with the Myc-tagged eIF3d (*myc-eIF3d-globin3’UTR*), untagged TurboID, and with *bfp-F’-nos3’UTR* mRNAs. Embryos of sample 1 and half of the embryos of sample 2 were additionally injected into the 1st blastomere with 100 µM biotin (Sigma-Aldrich) in 10 mM HEPES buffer. Biotin-injected embryos (sample 1 and one subset of sample 2 embryos, ~120 embryos per sample) were dechorionated manually and incubated at 31 °C in the 750 µM biotin (Sigma-Aldrich) solution in 0.3× Danieau’s for 6 h. The second half of the sample 2 embryos (also ~120 embryos) received no biotin treatment and served as a negative control. At ~6–7 hpf, all three samples were de-yolked by gentle dissociation with the enzyme-free Cell Dissociation Buffer (Gibco) by gentle pipetting and centrifuged twice at 1000× *g*. Afterward, pelleted cells were lysed with RIPA buffer (Thermo Fischer Scientific) supplemented with 1x protease inhibitor cocktail (Thermo Fischer Scientific). Embryo protein extracts were passed thrice through Vivaspin 500 (Sartorius) columns to remove excess free biotin according to the manufacturer’s instructions. Buffer was exchanged repeatedly with RIPA buffer (Thermo Fischer Scientific) supplemented with 1× Protease inhibitor cocktail (Thermo Fischer Scientific). The streptavidin pulldown was performed as described by (Xiong et al, 2021). In brief, protein extracts were mixed with Dynabeads MyOne Streptavidin C1 (Invitrogen). For that 200 µl bead slurry per sample was pre-washed with RIPA buffer (Thermo Fischer Scientific). The protein extract and beads mixtures in 2 ml LoBind tubes (Eppendorf) were incubated on a rotor wheel at 4 °C overnight (~ 16 h). The next day, the beads were separated from the protein extracts on a magnetic rack. The beads were washed with 1 ml of each of the following solutions: twice with RIPA buffer (Thermo Fischer Scientific) supplied with 1× Protease inhibitor cocktail (Thermo Fischer Scientific), once with 2% SDS in 50 mM Tris-HCl pH 7.5, once with 2 M urea in 10 mM Tris-HCl pH 8.0, and twice again in RIPA buffer (Thermo Fischer Scientific) without protease inhibitors. Washed beads were boiled in 60 µl of 2× LDS sample buffer (4X diluted to 2× with RIPA buffer (Thermo Fischer Scientific)) containing 2 mM biotin (Sigma-Aldrich) and 20 mM DTT (Sigma-Aldrich) at 95 °C for 10 min. Samples were resolved on Mini-PROTEAN TGX Stain-Free Precast Gels (Bio-Rad) and analyzed by western blotting using the 1:500 anti-Myc and 1:500 anti-HA antibodies. Details of the RNA constructs are presented in Table EV1, and the antibodies used in this study are listed in the Reagents and Tools table.

#### Statistical analysis

Statistical analyses were performed using Prism software (version 9.4.0, GraphPad). All quantifications were derived from a minimum of three independent biological replicates, with the data subsequently combined into a single dataset. Values are reported as the mean ± standard deviation unless otherwise noted. The Mann–Whitney *U* test was employed for pairwise comparisons, with *P* values below 0.05 deemed statistically significant. All embryo and cell analyses were conducted using unbiased approaches.

## Supplementary information

Table EV1

Peer Review File

Dataset EV1

Dataset EV2

Movie EV1

Expanded View Figures

## Data Availability

All source data are provided with this paper. The code is available on GitHub https://github.com/Julian-We/PPInet. The corresponding raw RNA-sequencing data have been deposited in the NCBI Sequence Read Archive under accession number PRJNA1357867. The source data of this paper are collected in the following database record: biostudies:S-SCDT-10_1038-S44319-026-00781-w.
