# Supplementary material for: A Nanos3-containing protein complex can activate RNA translation in primordial germ cells in vivo
Source: EMBO Rep. 2026 Apr 24;27(11):3078–98. doi: 10.1038/s44319-026-00781-w (PMC13260924; doi:10.1038/s44319-026-00781-w)
Supplement: Supplementary file 5 — Movie EV1 [file 44319_2026_781_MOESM5_ESM.zip › Movie EV1/Movie EV1_legend.pdf]

Movie EV1. Predicted structural conformation of the zebrafish Nanos3–Dnd1 complex based on AlphaFold2.

The model illustrates the predicted three-dimensional interaction between Nanos3 (pink) and Dnd1 (purple). Regions of high-confidence structural prediction are presented in blue, while intrinsically disordered or low-confidence regions are colored in orange. The animation highlights the most probable interface of interaction between the zinc finger 2 (ZF2) domain of Nanos3 and the RNA-binding domain (RBD) of Dnd1.
