# Supplementary material for: A Nanos3-containing protein complex can activate RNA translation in primordial germ cells in vivo
Source: EMBO Rep. 2026 Apr 24;27(11):3078–98. doi: 10.1038/s44319-026-00781-w (PMC13260924; doi:10.1038/s44319-026-00781-w)
Supplement: Supplementary file 6 — Expanded View Figures [file 44319_2026_781_MOESM6_ESM.pdf]

Expanded View Figures

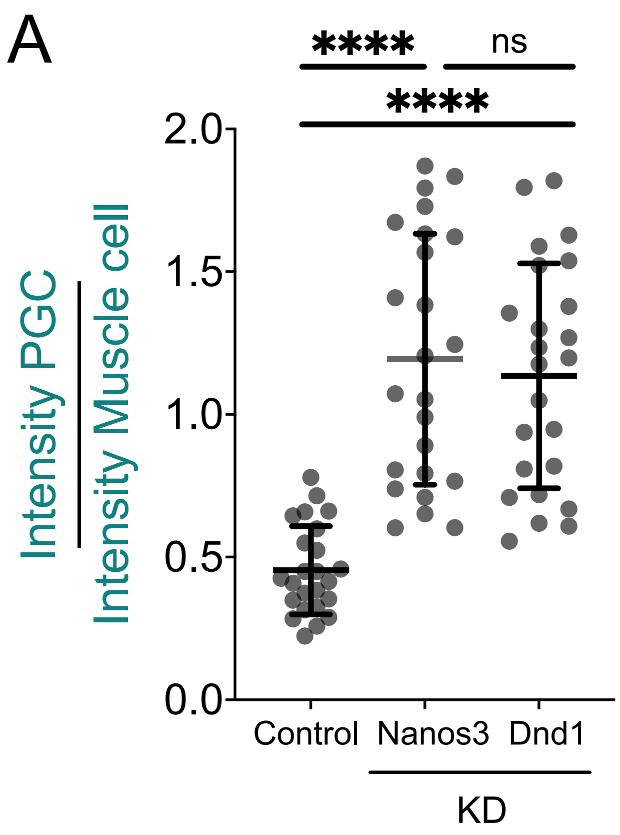

Figure EV1. Similarity between Nanos3 and Dnd1 knockdown phenotypes.

(**A**) Quantification of PGC-to-muscle TFP (teal fluorescent protein) fluorescence intensity ratios in Nanos3 knockdown (KD), Dnd1 KD, and control cells. See Fig. 1A,B for the experimental setup. Data represent mean ± SD ($n = 20$ cells per condition, $N = 3$ experiments). Mann–Whitney U test. ****$P < 0.0001$; ns not significant.

 © The Author(s)

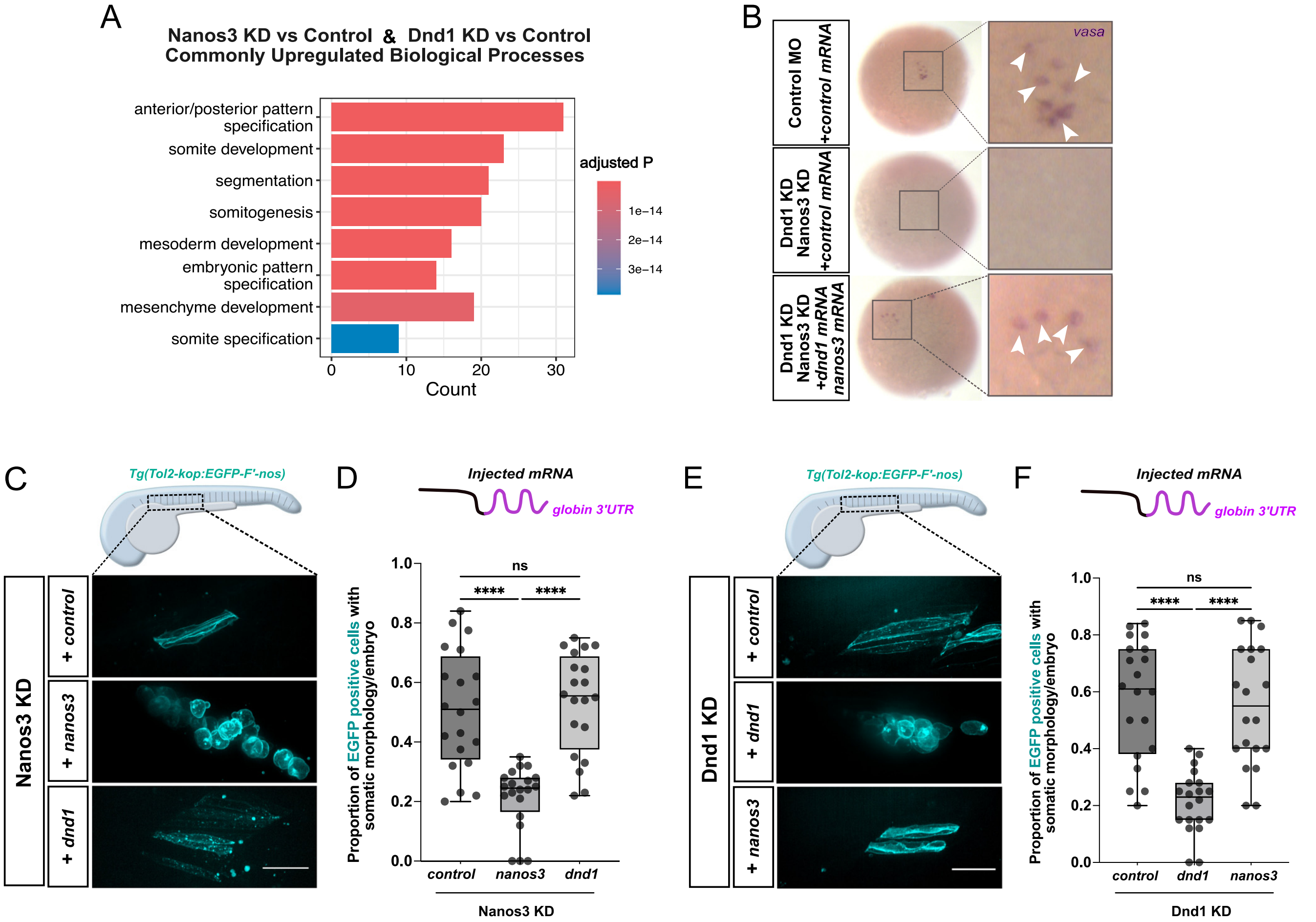

Figure EV2. Additional analysis demonstrating the cooperative function of Nanos3 and Dnd1 in PGC maintenance.

(**A**) Gene Ontology (GO) analysis showing biological processes enriched among transcripts upregulated both in Nanos3 KD and Dnd1 KD PGCs, compared to control. These processes include somatic differentiation and cellular reprogramming pathways. Bar length indicates the number of genes associated with each biological process. Statistical significance was assessed using a one-sided Fisher's exact test. Adjusted *P* values are represented by the color scale. (**B**) Whole-mount in situ hybridization employing the PGC marker *vasa* on control embryos, embryos depleted of Dnd1 and Nanos3 co-injected with control RNA, and embryos depleted of Dnd1 and Nanos3 co-injected with morpholino-resistant *nanos3* and *dnd1* RNAs. A rescue is observed when both RNAs are injected (lower panel). White arrowheads point at PGCs. (**C**, **D**) Images showing cell morphology and quantification of PGCs labeled with membrane-bound EGFP. The cells display a somatic-like morphology in Nanos3 KD embryos, with a rescue to the characteristic round PGC morphology observed only when morpholino-resistant *nanos3* RNA carrying a *globin* 3′UTR was injected, but not with *dnd1* RNA carrying a *globin* 3′UTR. Scale bar: 20 µm. (**E**, **F**) Images demonstrating cell morphology and quantification of PGCs labeled with membrane-bound EGFP displaying somatic-like morphology in Dnd1 KD embryos, with a rescue to the characteristic round PGC morphology observed only when morpholino-resistant *dnd1* RNA carrying a *globin* 3′UTR was injected, but not with *nanos3* RNA carrying a *globin* 3′UTR. Scale bar: 20 µm. (**C–F**) Data were obtained from $n = 20$ cells per condition from $N = 3$ independent experiments. Box plots show median (center line), interquartile range (box), and minimum to maximum values (whiskers). Individual data points are shown. Statistical significance was determined using the Mann–Whitney *U* test. $^{****}P < 0.0001$. Scale bar, 20 µm.

© The Author(s)

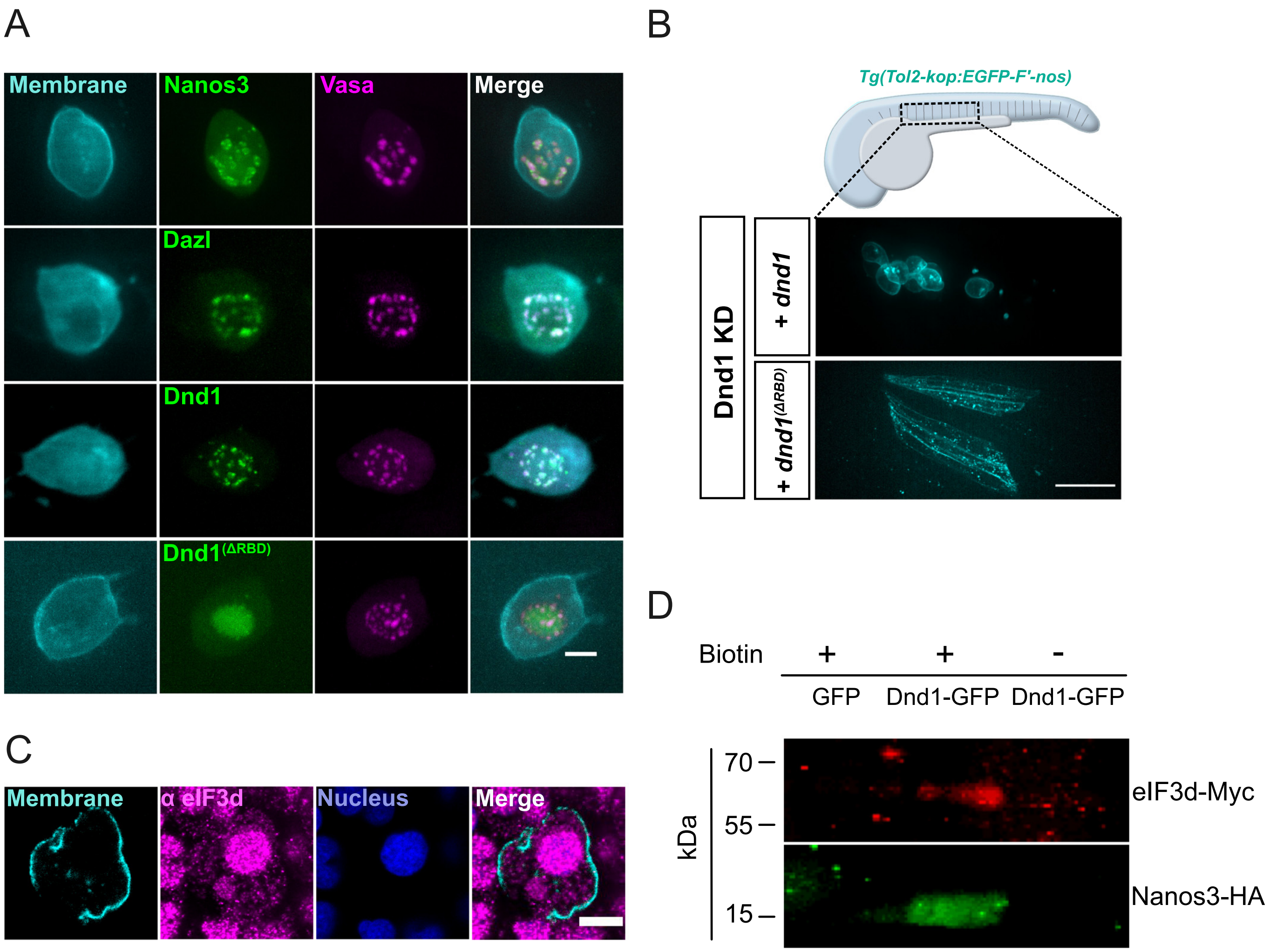

Figure EV3. Localization and function of PGC-expressed proteins, and interactions among them.

(**A**) Images showing wild-type localization of Nanos3, Dnd1, Dnd1$^{\Delta RBD}$, and Dazl GFP fusion proteins (green) relative to the cell membrane (cyan) and the germ cell granules (magenta). $n = 32$ cells per condition, $N = 3$ experiments. Scale bar: 6 µm. (**B**) Images showing the PGC morphology in transgenic zebrafish expressing membrane-bound EGFP at 24 h post fertilization. While Dnd1 can restore PGC round morphology and arrival at the gonad region in Dnd1 KD (upper panel), the Dnd1$^{\Delta RBD}$ form fails to do so. $n = 36$ cells per condition, $N = 3$ experiments. Scale bar: 20 µm. (**C**) Immunostaining of PGCs showing the endogenous eIF3d (magenta) localization. The merged image shows the presence of eIF3d in the nucleus and the cytoplasm. Scale bar: 2 µm. $n = 8$ cells, $N = 3$ experiments. (**D**) Proximity-dependent biotinylation assay. Transgenic (*Tg[bAct:mKate-p2A-TurboID-dGBP]*) embryos expressing cytoplasmic GFP or Dnd1-GFP were incubated in the presence or absence of biotin as indicated. Biotinylated proteins were isolated and analyzed by immunoblotting using anti-Myc (eIF3d-Myc, upper panel) and anti-HA (Nanos3-HA, lower panel) antibodies. Molecular weight markers (kDa) are indicated on the left.

© The Author(s)

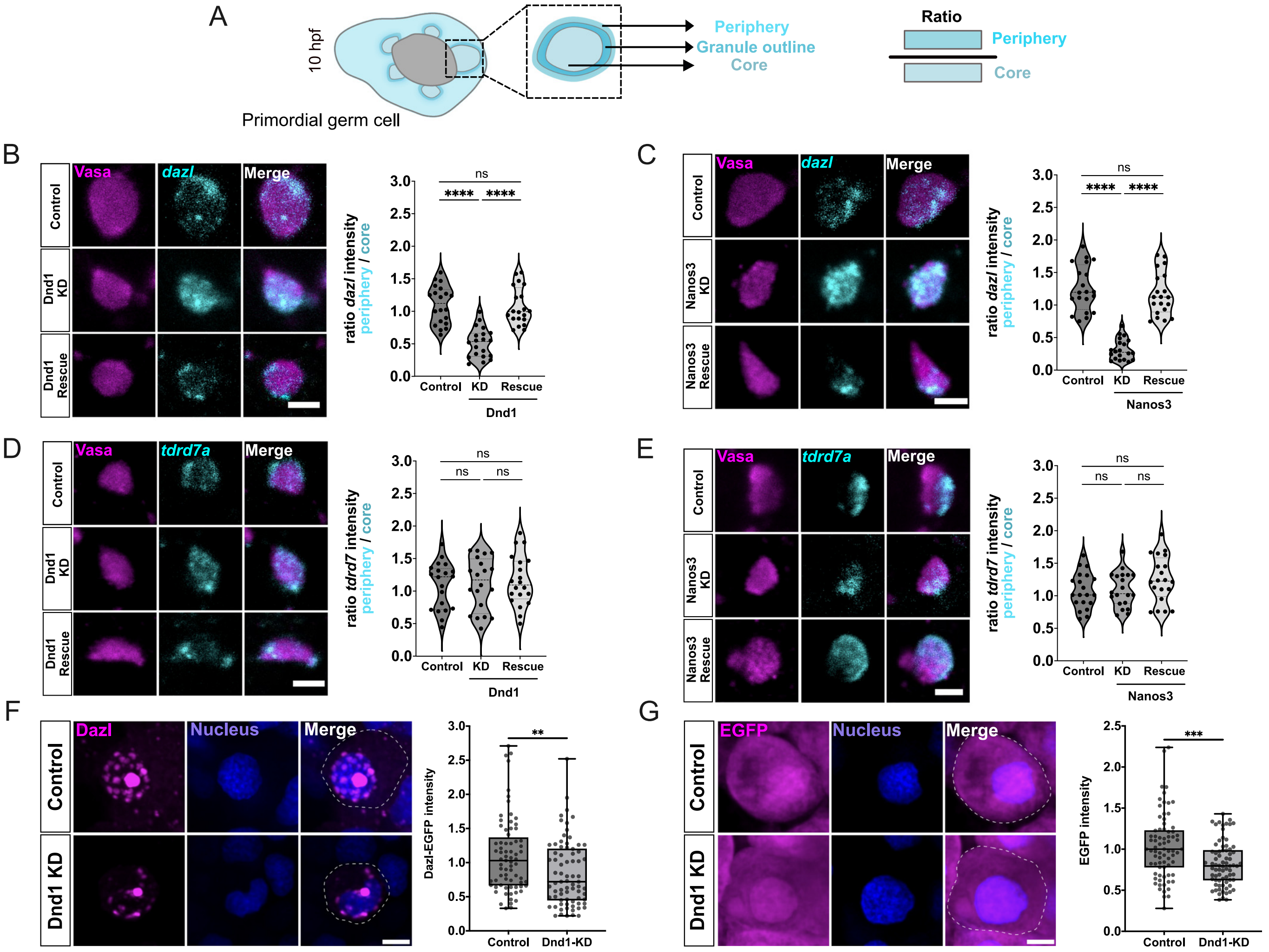

Figure EV4. The role of Nanos3 and Dnd1 in controlling the localization of other RNAs and protein production.

(**A**) Schematic illustration of a PGC with a magnified germ granule at 10 h post-fertilization (hpf). The inset presents the procedure used to calculate periphery-to-core intensity ratios to quantify the RNA distribution within and around the granule. (**B–E**) Images and quantification of different endogenous RNA localization (cyan) across the different experimental conditions. Anti-Vasa antibody (magenta) marks germ granules. Quantifications on the right of each panel present the periphery-to-core RNA intensity ratio. Scale bar: 2 µm. $n = 20$ cells per condition $N = 3$. Data represent mean ± SD. Mann–Whitney *U* test; *****P* < 0.0001; ns: not significant. (**F**) Images and quantification of Dazl protein levels in control and Dnd1 KD embryos, injected using the *dazl-EGFP-dazl 3′UTR* reporter RNA. Dotted lines indicate cell boundaries. Scale bar: 5 µm. Quantification was performed from $n = 75$ cells per condition obtained from $N = 3$ independent experiments. Box plots show median (center line), interquartile range (box), and minimum to maximum values (whiskers). Individual data points are shown. Statistical significance was determined using the Mann–Whitney *U* test. *****P* < 0.0001. (**G**) Images and quantification of EGFP protein levels in control and Dnd1 KD embryos, injected using the *EGFP-globin 3′UTR* reporter RNA. Dotted lines indicate cell boundaries. Scale bar, 5 µm. Quantification was performed from $n = 75$ cells per condition obtained from $N = 2$ independent experiments. Box plots show median (center line), interquartile range (box), and minimum to maximum values (whiskers). Individual data points are shown. Statistical significance was determined using the Mann–Whitney *U* test. *****P* < 0.0001.

© The Author(s)
