# Supplementary material for: A Nanos3-containing protein complex can activate RNA translation in primordial germ cells in vivo
Source: EMBO Rep. 2026 Apr 24;27(11):3078–98. doi: 10.1038/s44319-026-00781-w (PMC13260924; doi:10.1038/s44319-026-00781-w)
Supplement: Supplementary file 2 — Peer Review File [file 44319_2026_781_MOESM2_ESM.pdf]

EMBO reports

A Nanos3-containing protein complex can activate RNA translation in primordial germ cells in vivo

Antra Gupta, Arzu Basaran, Moritz Ophaus, Shantanu Madiwale, Martin Stehling, Michal Reichman-Fried, Katsiaryna Tarbashevich, and Erez Raz

Corresponding author(s): Erez Raz (erez.raz@uni-muenster.de)

Review Timeline:

Submission Date:	21st Jul 25
Editorial Decision:	1st Sep 25
Revision Received:	17th Feb 26
Editorial Decision:	24th Mar 26
Revision Received:	28th Mar 26
Accepted:	1st Apr 26

Editor: Martina Rembold

Transaction Report:

(Note: With the exception of the correction of typographical or spelling errors that could be a source of ambiguity, letters and reports are not edited. Depending on transfer agreements, referee reports obtained elsewhere may or may not be included in this compilation. Referee reports are anonymous unless the Referee chooses to sign their reports.)

1st Editorial Decision **1st Sep 2025**

Dear Prof. Raz

Thank you for the submission of your manuscript to EMBO reports. We have now received the full set of referee reports that is copied below.

As you will see, the referees acknowledge that the findings are interesting and that the conclusions are overall supported by the data presented but they also raise a number of concerns and have suggestions how to further strengthen the data, such as providing further evidence that Nanos3-Dnd1 increase translation (more broadly) or further data supporting complex formation of endogenous proteins.

Given the constructive comments and support by the referees, we would like to invite you to revise your manuscript with the understanding that the referee concerns (as detailed above and in their reports) must be fully addressed and their suggestions taken on board. Please address all referee concerns in a complete point-by-point response. Acceptance of the manuscript will depend on a positive outcome of a second round of review. It is EMBO Reports policy to allow a single round of revision only and acceptance or rejection of the manuscript will therefore depend on the completeness of your responses included in the next, final version of the manuscript.

We realize that it is difficult to revise to a specific deadline. In the interest of protecting the conceptual advance provided by the work, we recommend a revision within 3 months (December 1st). Please discuss the revision progress ahead of this time with the editor if you require more time to complete the revisions.

I am also happy to discuss the revision further via e-mail or a video call, if you wish.

You can either publish the study as a short report or as a full article. For short reports, the revised manuscript should not exceed 27,000 characters (including spaces but excluding materials & methods and references) and 5 main plus 5 expanded view figures. The results and discussion sections must further be combined, which will help to shorten the manuscript text by eliminating some redundancy that is inevitable when discussing the same experiments twice. For a normal article there are no length limitations, but it should have more than 5 main figures and the results and discussion sections must be separate. In both cases, the entire materials and methods must be included in the main manuscript file.

================

IMPORTANT NOTE:

We perform an initial quality control of all revised manuscripts before re-review. Your manuscript will FAIL this control and the handling will be delayed IN CASE the following APPLIES:

1) A data availability section providing access to data deposited in public databases is missing. If you have not deposited any data, please add a sentence to the data availability section that explains that.

2) Your manuscript contains statistics and error bars based on n=2. Please use scatter blots in these cases. No statistics should be calculated if n=2.

When submitting your revised manuscript, please carefully review the instructions that follow below. Failure to include requested items will delay the evaluation of your revision.

================

When submitting your revised manuscript, we will require:

1) a .docx formatted version of the manuscript text (including legends for main figures, EV figures and tables). Please make sure that the changes are highlighted to be clearly visible.

2) individual production quality figure files as .eps, .tif, .jpg (one file per figure).
Please download our Figure Preparation Guidelines (figure preparation pdf) from our Author Guidelines pages https://www.embopress.org/page/journal/14693178/authorguide for more info on how to prepare your figures.

3) a .docx formatted letter INCLUDING the reviewers' reports and your detailed point-by-point responses to their comments. As part of the EMBO Press transparent editorial process, the point-by-point response is part of the Review Process File (RPF), which will be published alongside your paper.

4) a complete author checklist, which you can download from our author guidelines (<https://www.embopress.org/page/journal/14693178/authorguide>). Please insert information in the checklist that is also reflected in the manuscript. The completed author checklist will also be part of the RPF.

5) Please note that all corresponding authors are required to supply an ORCID ID for their name upon submission of a revised manuscript (<https://orcid.org/>). Please find instructions on how to link your ORCID ID to your account in our manuscript tracking system in our Author guidelines
(<https://www.embopress.org/page/journal/14693178/authorguide#authorshipguidelines>)

6) We replaced Supplementary Information with Expanded View (EV) Figures and Tables that are collapsible/expandable online. A maximum of 5 EV Figures can be typeset. EV Figures should be cited as 'Figure EV1, Figure EV2" etc... in the text and their respective legends should be included in the main text after the legends of regular figures.

- For the figures that you do NOT wish to display as Expanded View figures, they should be bundled together with their legends in a single PDF file called *Appendix*, which should start with a short Table of Content. Appendix figures should be referred to in the main text as: "Appendix Figure S1, Appendix Figure S2" etc. See detailed instructions regarding expanded view here:
<https://www.embopress.org/page/journal/14693178/authorguide#expandedview>

- Additional Tables/Datasets should be labeled and referred to as Table EV1, Dataset EV1, etc. Legends have to be provided in a separate tab in case of .xls files. Alternatively, the legend can be supplied as a separate text file (README) and zipped together with the Table/Dataset file.

7) Before submitting your revision, primary datasets (and computer code, where appropriate) produced in this study need to be deposited in an appropriate public database (see <
https://www.embopress.org/page/journal/14693178/authorguide#dataavailability>).

Specifically, we would kindly ask you to provide public access to RNA seq dataset.

Please remember to provide a reviewer password if the datasets are not yet public.

The accession numbers and database should be listed in a formal "Data Availability " section (placed after Materials & Method) that follows the model below (see also < https://www.embopress.org/page/journal/14693178/authorguide#dataavailability>). Please note that the Data Availability Section is restricted to new primary data that are part of this study.

Data availability

The datasets (and computer code) produced in this study are available in the following databases:

- RNA-Seq data: Gene Expression Omnibus GSE46843 (https://www.ncbi.nlm.nih.gov/geo/query/acc.cgi?acc=GSE46843)
- [data type]: [name of the resource] [accession number/identifier/doi] ([URL or identifiers.org/DATABASE:ACCESSION])

*** Note - All links should resolve to a page where the data can be accessed. ***

8) At EMBO Press we ask authors to provide source data for the main manuscript figures. You will receive a separate email with instructions for providing source data with your revised manuscript, including how to upload and organize the files.

Additional information on source data and instruction on how to label the files are available
<https://www.embopress.org/page/journal/14693178/authorguide#sourcedata>

9) The journal requires a statement specifying whether or not authors have competing interests (defined as all potential or actual interests that could be perceived to influence the presentation or interpretation of an article). In case of competing interests, this must be specified in your disclosure statement. Further information: https://www.embopress.org/competing-interests

10) Figure legends and data quantification:
The following points must be specified in each figure legend:

- the name of the statistical test used to generate error bars and P values,
- the EXACT p-values,
- the number (n) of independent experiments (please specify technical or biological replicates) underlying each data point,
- the nature of the bars and error bars (s.d., s.e.m.)

- If the data are obtained from n {less than or equal to} 5, show the individual data points in addition to the SD or SEM.
- If the data are obtained from n {less than or equal to} 2, use scatter blots showing the individual data points.

Discussion of statistical methodology can be reported in the materials and methods section, but figure legends should contain a basic description of n, P and the test applied.

See also the guidelines for figure legend preparation:
https://www.embopress.org/page/journal/14693178/authorguide#figureformat

- Please also include scale bars in all microscopy images and define their size in the legend, not the image.

11) Our journal encourages inclusion of *data citations in the reference list* to directly cite datasets that were re-used and obtained from public databases. Data citations in the article text are distinct from normal bibliographical citations and should directly link to the database records from which the data can be accessed. In the main text, data citations are formatted as follows: "Data ref: Smith et al, 2001" or "Data ref: NCBI Sequence Read Archive PRJNA342805, 2017". In the Reference list, data citations must be labeled with "[DATASET]". A data reference must provide the database name, accession number/identifiers and a resolvable link to the landing page from which the data can be accessed at the end of the reference. Further instructions are available at <https://www.embopress.org/page/journal/14693178/authorguide#referencesformat>.

12) All Materials and Methods need to be described in the main text using our 'Structured Methods' format. According to this format, the Methods section includes a Reagents and Tools Table (listing key reagents, experimental models, software and relevant equipment and including their sources and relevant identifiers) followed by a Methods and Protocols section describing the methods, ideally using a step-by-step protocol format. The aim is to facilitate adoption of the methodologies across labs. Please download and fill our Reagents and Tools Table template (.docx), which you can find in our author guidelines: https://www.embopress.org/page/journal/14693178/authorguide#structuredmethods.
When submitting your revised manuscript, please do not include the Reagents and Tools Table in the Methods section of the manuscript but upload it as a separate file choosing the file type "Reagent Table".
An example of a Method paper with Structured Methods can be found here:
https://www.embopress.org/doi/10.15252/msb.20178071.

13) As part of the EMBO publication's Transparent Editorial Process, EMBO Reports publishes online a Review Process File to accompany accepted manuscripts. This File will be published in conjunction with your paper and will include the referee reports, your point-by-point response and all pertinent correspondence relating to the manuscript.

You are able to opt out of this by letting the editorial office know (emboreports@embo.org). If you do opt out, the Review Process File link will point to the following statement: "No Review Process File is available with this article, as the authors have chosen not to make the review process public in this case."

We would also welcome the submission of cover suggestions, or motifs to be used by our Graphics Illustrator in designing a cover.

I look forward to seeing a revised form of your manuscript when it is ready and please let me know if you have questions or comments regarding the revision.

Yours sincerely,

Martina Rembold, PhD
Senior Editor
EMBO reports

==================

Referee #1:

A Nanos3-containing protein complex activates RNA translation in primordial germ cells in vivo Antra Gupta1 , Arzu Melsa Basaran1 , Moritz Ophaus1 , Julian Wegner1 , Martin Stehling2 , Michal Reichman-Fried1 , Katsiaryna Tarbashevich1 , and Erez Raz1,2*

EMBOR-2025-62376V1-Merged_PDF

This manuscript reports a potentially impactful result of a new function of Nanos3 in the context of deadend1 in zebrafish. Instead of inhibiting mRNA retention of translation as is normally seen, these results suggest that Nanos3 and deadend1 form a complex with the translation factor EIf3d and promotes transition of specific mRNAs.
Overall, I conclude that the manuscript has logical progression in experimentation and conclusion building, and that it is nicely written. The figures are clear and where feasible, the results are quantitated showing individual datapoints.
Where I feel the manuscript falls short is as follows:
1) Is increased translation of select proteins identified and measured? The authors show specific effects on translation of Nanos3 and Dnd1 mRNAs, but a major premise of the work is how broad this selective increased translation is as a result of Nanos3/Dnd1 complexing with EIf3d. Is the increased translation of a cohort of proteins a hypothesis or a dataset?
2) The Discussion is underperforming - Nanos regulation of transition and RNA turnover has a ~60 year history ... and this distinction is mentioned in a portion of a sentence herein. "...nanos also recruits pumilio and CNOT complexes and other proteins important for translational inhibition and mRNA turnover..." - little is discussed on how this Nanos3 function finds distinction. How must competition between the different factors within the cells must shake out?
3) Expand on what is unique about Nanos3 from other nanos genes and how this new function might be so selective.
4) How does competition with endogenous eIF3d work? And was translation in those cells compromised? Do they have more EIf3d than potential somatic cells making this complex a key PGC potential?
5) Central to the premise and conclusions is that Nanos3/Dnd enhance translation of certain RNAs that they interact with, and that they accumulate in a translation rich environment in the cell. What is the evidence that selective new proteins are made as a result of the Nanos3/Dnd interaction? e.g. Not mRNA retention, but select protein synthesis and not just Nanos/Dnd?
6) Fig 6 is well underperforming but would be a great place to address some of these questions of selectivity in Nanos3/Dnd1 mRNA protein, competition with endogenous EIf3d, with other Nanos proteins, and how repositioning of the endogenous mRNAs in the cell promotes transition and mRNA retention.

Referee #2:

This manuscript presents a beautiful and compelling study uncovering that Nanos3 and Dnd1 form a complex that promotes translation of select germ-plasm RNAs at the periphery of PGC germ granules, creating a positive feedback loop that sustains germline fate in zebrafish. Knowledge on the general structure of germ granules as well as of the protein-protein and RNA-protein interactions in RNP complexes is extremely lacking, despite their critical and general importance. The evidence for Nanos3 acting as a translational activator are novel and important. The experiments throughout the work are elegantly designed and very-well controlled, and the data is very compelling. This is further emphasized by the fact that the work successfully answers questions that are extremely difficult to address in-vivo, and hence have long remained open.

The work thus provides a major and exciting advance in the understanding of germ granules and PGCs in zebrafish and in germline development, as well as more broadly in our knowledge on RNA-Protein complexes in other somatic types of granules and condensates.

Therefore, this work impressively provides timely and important findings, which in my opinion are highly suited for publication in EMBO Reports. I recommend acceptance with only few minor issues, detailed below.

Minor comments:

1. While demonstrating direct translational control in-vivo is difficult as the authors discuss and this reviewer well acknowledges, it would be nice to provide some evidence to strengthen this point. It would be nice to show correlation between translational machinery and the affected RNA under the different conditions within granules, like this group has shown previously with for example localization of the L10 60S ribosomal protein as a marker.

2. The study provides multiple strong evidence for the translational activation role of Nanos3. While this is compelling, the possibility that Nanos3 effects are mediated by an intermediate activator at least formally remains. That being said, experiments to address this in-vivo are nearly impossible to conduct, and should not be expected from the authors. Instead, the authors should only mention and discuss this formal possibility.

3. In Fig. 1A, the outline seems duplicated.

4. In Fig. 1C, should the quantification be muscle /PGC and not vice versa as shown?

5. In Fig. 1C, the indicated statistics are not clear - is N=2 refers to embryos or experiments? Please specify better, and include number of embryos in experiments.

6. There is a discrepancy in the mention of K100T or K200T in different text/figure parts, which should be corrected.

7. In Fig 6, "Fertility", and "Infertility, Teratoma formation" are not directly addressed in this work. I'd suggest removing these

terms from the summary figure (they seem more appropriate for a review). "Germline maintenance" and "Somatic reprograming" are sufficiently meaningful and important without those additions.

Referee #3:

This manuscript by Gupta et al. investigates the molecular mechanisms underlying Nanos3's role in safeguarding primordial germ cell (PGC) identity in zebrafish.
Key findings:
1. Nanos3 and Dnd1 form a complex in zebrafish PGCs.
2. This complex is essential for maintaining germline identity, preventing PGCs from transdifferentiating into somatic cells.
3. Dnd1 interacts with eIF3d.
4. The complex localizes target RNAs (nanos3, dnd1, dazl) to the periphery of phase-separated germ granules.
From these findings, the authors propose a novel role for Nanos3, in cooperation with Dead End1 (Dnd1), in promoting-rather than repressing-RNA translation, thereby maintaining germline fate through a positive regulatory loop.

General Comments
The interaction between Nanos3 and Dnd1 has been established in mice, and the exploration of a conserved mechanism across species is of significant interest. If this complex is indeed conserved but demonstrates divergent functions across species, it presents a particularly compelling avenue of investigation. However, although some of the data provide intriguing observations, they remain insufficient to fully substantiate the authors' claims. Additional experimental evidence is required before this work can be considered for publication in EMBO Reports.

Specific Issues
1. Given the similarity in phenotypic outcomes and transcriptomic changes observed in Nanos3 and Dnd1 knockdown experiments, it is reasonable to infer that Nanos3 and Dnd1 form a functional complex, even though the BiFC assay was performed in an overexpression system. By contrast, the evidence for Dnd1-eIF3d interaction relies solely on BiFC data. Overexpressed proteins may engage in non-physiological interactions or display aberrant subcellular localization. Notably, some Dnd1-eIF3d complexes appear to localize within the nucleus (Fig. 4B), which is unexpected, given that eIF3d is a translation initiation factor. Demonstrating interaction between endogenous proteins is therefore necessary. Alternatively, the authors should establish an experimental system in which exogenous Dnd1 or Nanos3 are expressed at near-physiological levels and repeat the assays.
2. The authors claim that the Nanos3-Dnd1 complex includes eIF3d based on Nanos3 localization near the YFP signals of the Dnd1-eIF3d complex (Fig. 4C) and the disappearance of these signals in Nanos3-KD PGCs (Fig. 4B). However, Nanos3 appears adjacent to, but not truly co-localized with, the YFP signals.
Moreover, in Nanos3-KD cells, Dnd1 protein levels are dramatically reduced (Fig. 5G), raising concerns about the robustness of the system. Independent evidence for the existence of a ternary complex in zebrafish PGCs is required.
3. If the Nanos3-Dnd1 complex promotes translation, some of its target genes should overlap with those downregulated in the KD experiments. It is essential to analyze whether such transcripts physically interact with the Nanos3-Dnd1 complex and whether they are localized to germ granule peripheries. In this regard, it would be important to clarify whether nanos3, dnd1, and dazl mRNAs fall within the set of genes showing reduced expression upon KD.
4. Page 9, Line 4: There is no image in Figure 4B showing Nanos3 co-localized with the Dnd1-eIF3d complex.
5. The RNA-seq results should be provided as supplementary data.
6. Figure 3A is presented without adequate explanation in the text.

We thank the reviewers for their time and very constructive feedback. In response, we have revised the text accordingly and performed additional experiments to address the issues raised. Below, we provide detailed point-by-point responses to each of the reviewer comments.

Referee #1:

This manuscript reports a potentially impactful result of a new function of Nanos3 in the context of deadend1 in zebrafish. Instead of inhibiting mRNA retention of translation as is normally seen, these results suggest that Nanos3 and deadend1 form a complex with the translation factor EIf3d and promotes transition of specific mRNAs. Overall, I conclude that the manuscript has logical progression in experimentation and conclusion building, and that it is nicely written. The figures are clear and where feasible, the results are quantitated showing individual datapoints. Where I feel the manuscript falls short is as follows:
1) Is increased translation of select proteins identified and measured? The authors show specific effects on translation of Nanos3 and Dnd1 mRNAs, but a major premise of the work is how broad this selective increased translation is as a result of Nanos3/Dnd1 complexing with EIf3d. Is the increased translation of a cohort of proteins a hypothesis or a dataset?

We thank the reviewer for raising this point. In Figure 6 of the revised manuscript we show that knockdown of Dnd1 leads to a strong reduction in Nanos3 protein levels (approximately 75%), and that Nanos3 knockdown lowers the level of Dnd1 protein (approximately 30%), suggesting interdependence of their translation.

Nevertheless, in line with the reviewer's view, we consider that this mechanism could apply to additional germline RNAs. Our data indeed suggests that the positive impact on translation extends beyond Nanos3 and Dnd1, likely reflecting on both direct targets of the complex, as well as indirect effects. This conclusion is supported by two sets of experiments described below.

First, we now include an additional experiment in a new version of Figure EV4 (panels F,G). Here, we show that the level of the germline-expressed protein Dazl is strongly reduced following Dnd1 knockdown, suggesting direct interaction that is in line with the "internalisation" of the *dazl* RNA into the granule core presented in Figure EV4B. GFP reporter linked to a globin UTR is affected as well, likely representing an indirect effect. We describe this in the revised text as follows: "Additionally, we observed decreased Dazl protein levels following Dnd1 depletion (approximately 30%) (Fig. EV4F), suggesting that the Nanos3–Dnd1 complex supports the translation of other germline determinants. Whereas the *globin 3'UTR*-linked GFP reporter showed a significant but comparatively small decrease upon depletion of Dnd1 (approximately 20%) (Fig. EV4G)."

Second, ectopic expression of Nanos3 and Dnd1 throughout the embryo (eIF3d is ubiquitously expressed in the early embryo) leads to the transformation of all the cells of the embryo into PGCs. In those cells, we observe the formation of the complex and, importantly, when these

induced PGCS were transplanted into hosts depleted of endogenous PGCs they give rise to gametes and make the hosts fertile. Similar findings have been reported for Medaka (Nishimura & Fujimoto, 2025), and we are currently investigating this phenomenon further. We refer to this point in the Introduction as “Importantly, in medaka, ectopic co-expression of Nanos3 and Dead End1 (Dnd1) in somatic cells induces the formation of PGC-like cells that successfully migrate to the gonads and differentiate into functional gametes (Nishimura & Fujimoto, 2025), underscoring Nanos3 as a central regulator of germ cell fate.”

Figure for referee with unpublished data and its description has been removed upon request by the authors.

2) The Discussion is underperforming - Nanos regulation of transition and RNA turnover has a ~60 year history ... and this distinction is mentioned in a portion of a sentence herein.

"...nanos also recruits pumilio and CNOT complexes and other proteins important for translational inhibition and mRNA turnover..." - little is discussed on how this Nanos3 function finds distinction. How must competition between the different factors within the cells must shake out?

In response to this point, we have now expanded the Discussion to place our findings in the context of the established roles of Nanos proteins in translational repression and RNA turnover, and to explain how the activity of Nanos3 described here differs from its previously described functions. We now discuss how interactions with specific cofactors and the cellular environment can influence Nanos3 function, providing a basis for distinct regulatory outcomes within the same cell. Specifically, we now state: "The role of Nanos3 described here differs from the canonical function of Nanos, which has been shown to function as a translational repressor acting with Pumilio to inhibit Hunchback protein production (Irish et al, 1989; Sonoda & Wharton, 1999). Nanos3 is the only known isoform to be expressed in zebrafish PGCs. During early embryogenesis, we find Nanos3 in complex with Dnd1, where it contributes to translational activation. Dnd1 has been reported to promote RNA stability and translation (Kedde et al, 2007; Westerich et al, 2023b), and its association with Nanos3 suggests that when bound to Dnd1, Nanos3 can contribute to enhanced stability and translation activation of target RNAs. Similar context-dependent function has been described for other RNA-binding proteins, including PUF family members, whose activity is shaped by cellular context, RNA targets and binding partners (Quenault et al, 2011). Together, these observations support a view of Nanos3 as a regulator that can function as either a repressor or an activator depending on its associated cofactors (e.g. CNOT complex (Suzuki et al, 2014) or Dnd1 respectively). While our data identify Dnd1 as a key determinant of this activity in vivo, the involvement of additional intermediate components cannot be excluded, and resolving the underlying mechanisms will require complementary in vitro biochemical approaches in other systems.''

3) Expand on what is unique about Nanos3 from other nanos genes and how this new function might be so selective.

We thank the referee for this comment. We did not discuss this point in the submitted version of the paper due to space restrictions in the Journal, and because we did not directly study other Nanos isoforms in detail. Based on DNA sequence analysis, Zebrafish has three Nanos orthologs: Nanos1, Nanos2, and Nanos3. These isoforms differ in their time of expression, and expression patterns. Nanos2 is expressed in adult gonads, particularly in the ovary, where it is required to prevent premature differentiation of germ cells (Beer & Draper, 2013; Cao et al, 2019). The expression pattern of Nanos1 was not described.

We also now note in the Discussion that Nanos3 is the only Nanos ortholog known to be endogenously expressed in primordial germ cells at this developmental stage.

Figure for referee with unpublished data and its description has been removed upon request by the authors.

4) How does competition with endogenous eIF3d work? And was translation in those cells compromised? Do they have more EIf3d than potential somatic cells making this complex a key PGC potential?

We thank the reviewer for this question. According to in situ hybridization data from (Thisse & Thisse, 2008), during the stages we study eIF3d is expressed uniformly. We now mention this point in the text. We observed no signs of compromised translation in the experiments involving eIF3d (BiFC experiments presented in Figure 4B). Since the Nanos3–Dnd1 complex is expressed in PGCs, it is not the higher level of eIF3d in the PGCs, but rather the presence of the Nanos3–Dnd1 complex that enables selective engagement with eIF3d and activation of germ cell-specific translation.

5) Central to the premise and conclusions is that Nanos3/Dnd enhance translation of certain RNAs that they interact with, and that they accumulate in a translation rich environment in the cell. What is the evidence that selective new proteins are made as a result of the Nanos3/Dnd interaction? e.g. Not mRNA retention, but select protein synthesis and not just Nanos/Dnd?

We thank the reviewer for this question. Based on the interaction of the Nanos3–Dnd1 protein complex with a translation initiation factor, its binding to *nanos3* and *dnd1* RNAs, and the effect on translation of each other's maternally-provided RNA, we propose that Nanos3 and Dnd1 positively regulate their own protein levels. Indeed, we have not systematically assessed the extent to which this mechanism operates directly on additional RNAs. Addressing this would require quantitative analysis on protein levels combined with the screening for RNAs physically associated with the Nanos3–Dnd1 complex, followed by functional assays comparable to those performed for *nanos3* and *dnd1*.Without such biochemical data, it would be difficult to distinguish direct effects of the complex from secondary consequences in our in vivo system.

Nevertheless, as stated in our response to Comment 1, we examined whether the Nanos3–Dnd1 complex could, in principle affect the translation of additional germline targets. In Figure EV4F, we now show that the protein level of Dazl, a germline-specific factor, is strongly reduced upon Dnd1 knockdown. This reduction is correlated with the change in the distribution pattern of *dazl* RNA shown in Figure EV4B, where its level is reduced at the granule periphery.

To further investigate this, we examined the distribution of the ribosomal marker L10a and found that it remains enriched at the periphery of the granule under knockdown conditions (Figure 6B). This indicates that the translation machinery is still present outside the germ granule, but *dazl* RNA is no longer in a position to engage with it. These results suggest that the Nanos3–Dnd1 complex plays a key role in positioning specific RNAs within a translation-competent environment, supporting the synthesis of germline proteins that are essential for maintaining cell identity.

6) Fig 6 is well underperforming but would be a great place to address some of these questions of selectivity in Nanos3/Dnd1 mRNA protein, competition with endogenous EIf3d, with other Nanos proteins, and how repositioning of the endogenous mRNAs in the cell promotes transition and mRNA retention.

We thank the reviewer for this suggestion. In response, we have revised the schematic model, now presented as Figure 7, to better reflect the findings of the study and to incorporate the referee's comments regarding selectivity and potential additional targets. We now use the term "postulated" when referring to possible other targets.

Regarding the competition with other Nanos proteins, we now address this point in the Discussion, where we state that nanos2 is not expressed in the PGCs whereas nanos3 is the ortholog active at this stage. We hope that the revised model provides a clearer representation

of the RNA–protein interactions and illustrates the role of the Nanos3–Dnd1 complex in translation, particularly concerning RNA positioning relative to the translation machinery.

Referee #2:

This manuscript presents a beautiful and compelling study uncovering that Nanos3 and Dnd1 form a complex that promotes translation of select germ-plasm RNAs at the periphery of PGC germ granules, creating a positive feedback loop that sustains germline fate in zebrafish. Knowledge on the general structure of germ granules as well as of the protein-protein and RNA-protein interactions in RNP complexes is extremely lacking, despite their critical and general importance. The evidence for Nanos3 acting as a translational activator are novel and important. The experiments throughout the work are elegantly designed and very-well controlled, and the data is very compelling. This is further emphasized by the fact that the work successfully answers questions that are extremely difficult to address in-vivo, and hence have long remained open.

The work thus provides a major and exciting advance in the understanding of germ granules and PGCs in zebrafish and in germline development, as well as more broadly in our knowledge on RNA-Protein complexes in other somatic types of granules and condensates.

Therefore, this work impressively provides timely and important findings, which in my opinion are highly suited for publication in EMBO Reports. I recommend acceptance with only few minor issues, detailed below.

Minor comments:

1. While demonstrating direct translational control in-vivo is difficult as the authors discuss and this reviewer well acknowledges, it would be nice to provide some evidence to strengthen this point. It would be nice to show correlation between translational machinery and the affected RNA under the different conditions within granules, like this group has shown previously with for example localization of the L10 60S ribosomal protein as a marker.

We thank the reviewer for the constructive suggestion. As requested, we now include data in Figure 6B showing the localization of L10a, a component of the 60S ribosomal subunit and a marker of the translational machinery. We observe that L10a at the periphery of the germ granule under all conditions tested. However, following Nanos3 knockdown, *dnd1* RNA accumulates at the granule core, away from the ribosomal protein. This spatial uncoupling supports the idea that translation is reduced when RNAs are no longer positioned near the "translation-competent environment". These findings further support our model in which spatial RNA positioning within the granule contributes to translational control.

2. The study provides multiple strong evidence for the translational activation role of Nanos3. While this is compelling, the possibility that Nanos3 effects are mediated by an intermediate activator at least formally remains. That being said, experiments to address this in-vivo are nearly impossible to conduct, and should not be expected from the authors. Instead, the authors should only mention and discuss this formal possibility.

We thank the reviewer for acknowledging the strength of the evidence supporting the role of Nanos3 in translational activation. As the reviewer notes, formally excluding the involvement of intermediate activators in an in vivo setting would indeed be nearly impossible, since we have no control over the presence of factors we do not know of. As suggested, we acknowledge this possibility in the Discussion. We state "The role of Nanos3 described here differs from the canonical function of Nanos, which has been shown to function as a translational repressor acting with Pumilio to inhibit Hunchback protein production (Irish et al, 1989; Sonoda & Wharton, 1999). Nanos3 is the only known isoform to be expressed in zebrafish PGCs. During early embryogenesis, we find Nanos3 in complex with Dnd1, where it contributes to translational activation. Dnd1 has been reported to promote RNA stability and translation (Kedde et al, 2007; Westerich et al, 2023b), and its association with Nanos3 suggests that when bound to Dnd1, Nanos3 can contribute to enhanced stability and translation activation of target RNAs. Similar context-dependent function has been described for other RNA-binding proteins, including PUF family members, whose activity is shaped by cellular context, RNA targets and binding partners (Quenault et al, 2011). Together, these observations support a view of Nanos3 as a regulator that can function as either a repressor or an activator depending on its associated cofactors (e.g. CNOT complex (Suzuki et al, 2014) or Dnd1 respectively). While our data identify Dnd1 as a key determinant of this activity in vivo, the involvement of additional intermediate components cannot be excluded, and resolving the underlying mechanisms will require complementary in vitro biochemical approaches in other systems.''

3. In Fig. 1A, the outline seems duplicated.
We thank the reviewer for noticing this. The duplicated outline has been corrected in the revised figure.

4. In Fig. 1C, should the quantification be muscle /PGC and not vice versa as shown?

We thank the reviewer for this question. We chose to present the quantification as the ratio of the signal intensity in the PGCs divided by the intensity in the muscle tissue in order to directly illustrate the increase in the expression of the muscle reporter in the PGCs upon Nanos3 knockdown. This way, the value increases. We could change the presentation to the intensity in muscle cell divided by PGCs if the referee considers it clearer.

5. In Fig. 1C, the indicated statistics are not clear - is N=2 refers to embryos or experiments? Please specify better, and include number of embryos in experiments.
In the submitted version "N" represented the number of independent biological replicates, and "n" referred to the number of individual germ cells analyzed. Each germ cell was derived

from a separate embryo. We have updated the figure legend to clarify this, stating "Data represent mean ± SD (n = 24 cells per condition, N = 3 experiments). Mann–Whitney U test, ****P < 0.0001."

6. There is a discrepancy in the mention of K100T or K200T in different text/figure parts, which should be corrected.

We thank the reviewer for noticing this mistake. The correct designation is K^{200T}, and we have corrected the figure labels accordingly.

7. In Fig 6, "Fertility", and "Infertility, Teratoma formation" are not directly addressed in this work. I'd suggest removing these terms from the summary figure (they seem more appropriate for a review). "Germline maintenance" and "Somatic reprograming" are sufficiently meaningful and important without those additions.
We fully agree with the reviewer. Accordingly, the terms "Fertility" and "Infertility, Teratoma formation" have been omitted. To focus on the key findings of this study, we have also removed the labels "Germline maintenance" and "Somatic reprogramming." The updated model, now presented as Figure 7, shows that Nanos3 and Dnd1 proteins act together in a complex, and this complex serves as a core regulatory unit that activates of germline RNA translation, which is essential for promoting PGC fate.

Referee #3:

This manuscript by Gupta et al. investigates the molecular mechanisms underlying Nanos3's role in safeguarding primordial germ cell (PGC) identity in zebrafish.
Key findings:
1. Nanos3 and Dnd1 form a complex in zebrafish PGCs.
2. This complex is essential for maintaining germline identity, preventing PGCs from transdifferentiating into somatic cells.
3. Dnd1 interacts with eIF3d.
4. The complex localizes target RNAs (nanos3, dnd1, dazl) to the periphery of phase-separated germ granules.
From these findings, the authors propose a novel role for Nanos3, in cooperation with Dead End1 (Dnd1), in promoting-rather than repressing-RNA translation, thereby maintaining germline fate through a positive regulatory loop.

General Comments

The interaction between Nanos3 and Dnd1 has been established in mice, and the exploration of a conserved mechanism across species is of significant interest. If this

complex is indeed conserved but demonstrates divergent functions across species, it presents a particularly compelling avenue of investigation. However, although some of the data provide intriguing observations, they remain insufficient to fully substantiate the authors' claims. Additional experimental evidence is required before this work can be considered for publication in EMBO Reports.

Specific Issues

1. Given the similarity in phenotypic outcomes and transcriptomic changes observed in Nanos3 and Dnd1 knockdown experiments, it is reasonable to infer that Nanos3 and Dnd1 form a functional complex, even though the BiFC assay was performed in an overexpression system. By contrast, the evidence for Dnd1-eIF3d interaction relies solely on BiFC data. Overexpressed proteins may engage in non-physiological interactions or display aberrant subcellular localization. Notably, some Dnd1-eIF3d complexes appear to localize within the nucleus (Fig. 4B), which is unexpected, given that eIF3d is a translation initiation factor. Demonstrating interaction between endogenous proteins is therefore necessary. Alternatively, the authors should establish an experimental system in which exogenous Dnd1 or Nanos3 are expressed at near-physiological levels and repeat the assays.

We thank the reviewer for raising the concern of potential overexpression artifacts in the BiFC assay, which is an important concern we addressed when setting up the experiment, but did not describe in the submitted paper. All BiFC experiments were conducted using a concentration of 100 ng/µl of RNA for each construct. This amount of RNA represents the minimal concentration required to obtain germ cells under conditions of knocking down Dnd1 or Nanos3 (see example below for Dnd1 KD). Higher RNA amounts were avoided to reduce the likelihood of non-physiological interactions. In this context, it is important to note that zebrafish PGCs initially show high variability in the amount of germ plasm they acquire. As a result, some cells develop as PGCs as a result of “just passing the threshold”, while others express the relevant factors at levels that are around 3-4 times higher. Accordingly, due to the possibility of artifacts stemming from BiFC signal due to overexpression, we omitted a panel from Figure 3B in the original submission, where overexpression was used on purpose (lower row in this figure). The message of the omitted panel is identical to that depicted in Figure 3D, i.e. relocalization of the mutant Dnd1 protein into the cytoplasm, but not by assaying the BiFC signal. We now mention in the methods section “All BiFC experiments were performed using the minimal RNA concentration required to allow development of the primordial germ cells.”

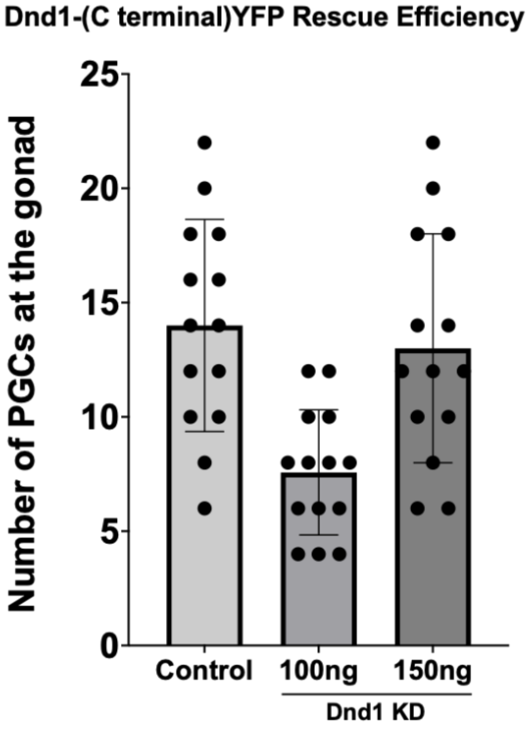

Rescue of Dnd1 knockdown by YFP(C-terminal)-Dnd1 Quantification of primordial germ cells (PGCs) at the gonad region in control embryos (injected with control morpholino and *control* RNA), and in Dnd1-depleted embryos rescued with morpholino resistant *YFP(C-terminal)-dnd1* RNA at the indicated concentrations. Control embryos show the normal PGC numbers at the gonad. Injection of 2nl of a 100 ng/µl *YFP(C-terminal)-dnd1* RNA results in partial rescue, whereas injection of m150 ng/µl *YFP(C-terminal)-dnd1* RNA restores PGC numbers to levels comparable to controls. Each dot represents a single primordial germ cell, bars indicate the mean, and error bars represent standard deviation.

Regarding the localization of the Dnd1–eIF3d BiFC signal in the nucleus, we now present data showing the distribution of endogenous eIF3d as observed by antibody staining in Figure EV3C. eIF3d is present in both the cytoplasm and nucleus of germ cells. These findings is consistent with the idea that the regions of interaction observed in the BiFC assay fit to endogenous localization patterns.

We do not know if the localization of the proteins in the nucleus signifies a role (e.g., associating with RNA in the nucleus). We observe Nanos3 in this compartment, as well as eIF3d (using an antibody). In non-manipulated cells, the wild-type Dnd1 protein is found preferentially in the germ granules, but is also detected within the nucleus (Fig EV3A and Figure 1 in (Weidinger et al, 2003)). Dnd1 protein that is defective in interaction with RNA or Nanos3 shows a strong increase in localisation to the nucleus (Figures 3D and EV3A), suggesting an affinity to this part of the cell.

In addition to the in vivo BIFC assay, we have also employed Alphafold2-based in silico assay, which predicts Nanos3 interacting with Dnd1 with high degrees of confidence (Figure 3A and Figure 4A). To validate the Nanos3–Dnd1 interaction employing an additional independent

method, we conducted two new experiments presented in Figure 3C and Figure EV3D. Here, we fused Dnd1 to a kinesin motor domain to relocalize it to discrete kinesin-positive regions. We found that Nanos3, which is distributed in the cytoplasm, germ granules, and nucleus of non-manipulated cells, is recruited to spots where the Dnd1-Kif5a is localised, with a corresponding reduction of signal in the cytoplasm and the nucleus. Second, as presented in detail in the response to point 2, we have also verified the interaction employing the Proximity-dependent biotinylation assay.

Together, the findings from Alphafold2, BiFC assay, and now Kinesin-mediated relocalization and Proximity-dependent biotinylation assay indicate that a Nanos3–Dnd1 complex forms in zebrafish primordial germ cells and that its localization and interactions are consistent with physiological expression patterns.

2. The authors claim that the Nanos3-Dnd1 complex includes eIF3d based on Nanos3 localization near the YFP signals of the Dnd1-eIF3d complex (Fig. 4C) and the disappearance of these signals in Nanos3-KD PGCs (Fig. 4B). However, Nanos3 appears adjacent to, but not truly co-localized with, the YFP signals. Moreover, in Nanos3-KD cells, Dnd1 protein levels are dramatically reduced (Fig. 5G), raising concerns about the robustness of the system. Independent evidence for the existence of a ternary complex in zebrafish PGCs is required.

We thank the reviewer for observing this technical mistake. The apparent separation between Nanos3 and the Dnd1–eIF3d BiFC signal resulted from a directional shift in one visualisation channel during image overlay. This has now been corrected, and the revised images in Figures 4B and 4C confirm that at the resolution level of spinning disk confocal microscopy, Nanos3 co-localizes with the Dnd1–eIF3d BiFC signal in primordial germ cells.

According to the results presented in this study and the model we propose, the Nanos3–Dnd1 complex enhances the translation of *nanos3* and *dnd1* RNAs. Consistently, we have previously shown that in the absence of Dnd1, Nanos3 protein level is reduced (Westerich et al, 2023) and present a similar result in this manuscript (Figure 6E). Since Nanos3 and Dnd1 form a complex, we therefore expect reciprocal dependency. Indeed, depletion of Nanos3 results in a strong reduction of Dnd1 protein levels, as shown in the current version of the manuscript in Figure 6D. These findings highlight a functional positive feedback loop, in which the Nanos3–Dnd1 complex supports Nanos3 and Dnd1 protein production and maintains *dnd1* RNA in a translationally active zone, outside the granule.

Regarding the interaction among the three proteins, we consider the BiFC assay to be a sensitive assay that reports on transient interactions and allows visualization of where these interactions occur in the live organism. Furthermore, as an additional assay, we conducted a Proximity-dependent biotinylation assay (Xiong et al, 2021). Using this method, we show that Nanos3, Dnd1, and eIF3d are localized in close proximity and can be pulled down (EV3D). In Transgenic zebrafish *Tg[bAct:mKate-p2A-TurboID-dGBP]* embryos, we expressed tagged versions of Nanos3, Dnd1 and eIF3d (HA, GFP, and Myc respectively), together with biotin and an anti-GFP nanobody linked to the TurboID. As presented in the methods section, we

pulled down biotinylated proteins using Dnd1-GFP as bait, and detected the tagged Nanos3 and eIF3d proteins in the pulled down fraction, which were missing in the negative controls.

Taken together, the corrected localization data, the BiFC assays, and the proximity-based interaction assay provide independent support for the existence of a ternary Nanos3–Dnd1–eIF3d complex in zebrafish primordial germ cells.

3. If the Nanos3-Dnd1 complex promotes translation, some of its target genes should overlap with those downregulated in the KD experiments. It is essential to analyze whether such transcripts physically interact with the Nanos3-Dnd1 complex and whether they are localized to germ granule peripheries. In this regard, it would be important to clarify whether nanos3, dnd1, and dazl mRNAs fall within the set of genes showing reduced expression upon KD.

We thank the reviewer for this comment and present our response and also an additional experiment we conducted. In our model, knocking down Dnd1 or Nanos3 leads to a reduction in translation of their RNAs rather than causing an immediate reduction in the level of the RNAs themselves. Loss of translation precedes the reduction in the level of germ-cell transcripts and the increase in the level of somatic transcripts. As previously shown, germ cells lacking Dnd1 or Nanos3 adopt a somatic transcriptional program, and somatic cells actively degrade germline RNAs (Köprunner et al, 2001; Wolke et al, 2002; Gross-Thebing et al, 2017; Westerich et al, 2023).

Consistent with this framework, RNAs that are no longer efficiently translated due to loss of Nanos3 or Dnd1 translocate into the granule core. As development progresses, germ granule structure disintegrates in Dnd1/Nanos3 knockdown PGCs, a reduction in transcript levels can be observed. Thus, the Nanos3–Dnd1 complex maintains target RNAs in regions of the cell that are permissive for translation, rather than directly controlling the total RNA levels (see Figure 5A,B in Westerich et al, 2023).

We agree that it is important to demonstrate physical association between the complex and its target RNAs. In the revised manuscript, we therefore present RNA immunoprecipitation experiments using tagged Nanos3. Indeed, we observe *nanos3* and *dnd1* RNAs in the precipitated fraction in this assay (Figure 5F in the current version of the manuscript), demonstrating that these transcripts physically associate with the Nanos3–Dnd1 complex in vivo. Together with the RNA localization and protein expression data, these results support a model in which translation of specific germline RNAs is regulated through their interaction with the Nanos3–Dnd1 complex.

4. Page 9, Line 4: There is no image in Figure 4B showing Nanos3 co-localized with the Dnd1-eIF3d complex.

We thank the reviewer for this comment. In the answer to comment 2, which relates to the same panel (Figure 4B), we have corrected a shift between the imaging channels such that the co-localisation between Nanos3 and the Dnd1–eIF3d complex is observed.

5. The RNA-seq results should be provided as supplementary data.

The RNA sequencing results relevant for Figure 1 are now presented in Table S1. The corresponding raw RNA sequencing data have been deposited in the NCBI Sequence Read Archive under accession number PRJNA1357867, as indicated in the Data Availability section.

6. Figure 3A is presented without adequate explanation in the text.
We thank the reviewer for pointing this out. The description of Figure 3A has been revised and in the current version it is stated " To explore this, we employed AlphaFold2 structural modeling (Jumper et al, 2021) to examine the potential associations between the two proteins. Indeed, this in silico analysis predicted an interaction mediated by the zinc finger 2 (ZF2) domain of Nanos3 and the RNA-binding domain (RBD) of Dnd1 (Fig.3A, Movie S1)."

Dear Prof. Raz

Thank you for the submission of your revised manuscript to EMBO reports. We have now received the reports of the referees that were asked to assess it.

As you will see, both referees are very positive about the study and request only minor changes to clarify text and figures.

From the editorial side, there are also a few things that we need before we can proceed with the official acceptance of your study.

- The manuscript sections should be in the following order: Title page - Abstract & Keywords - Introduction - Results - Discussion - Methods - Data Availability - Acknowledgments - Disclosure Statement & Competing Interests - References - Figure Legends - (Main Tables with legends if applicable) - Expanded View Figure Legends.

- Declaration of Interests needs to be renamed to Disclosure and Competing Interests Statement.

- Tables S1 and S2 in the Appendix are complex datasets. Please upload them individually as .xls files called Dataset EV#. The legend is part of the .xls file and placed in a separate tab. Please update all callouts in the paper accordingly. The Appendix is thus obsolete as the movie legend is part of the movie zip file. See next point.

- You state in the methods section that antibodies are listed in Table S3. I think this should refer to the Reagents and Tools table.

- Table S4 oligonucleotides should be Table EV1. Please update the table name, the legend and the callout in the Reagents and Tools table.

(With this you should have Dataset EV1 and EV2, I believe, Table EV1, Reagents and Tools table).

- Movie: the legend should be removed from the manuscript and provided as a text file and then zipped up with the movie; the correct nomenclature should be Movie EV1 in all places (source file names, titles in the system, legend, callout in the manuscript).

- The Data availability section should only refer to the deposited data. Please remove the statement "Further information or requests for resources and reagents should be directed to the Lead Contact, Erez Raz." Moreover, we would need an URL that resolves to the dataset PRJNA1357867.

- The code on github can also be listed in the Data availability section (The code is available on GitHub (https://github.com/Julian-We/PPInet).

- The Nomenclature of EV figure legends needs to be Figure EV1, etc. instead of Expanded View EV1.

- Regarding the Author Contributions, we now use CRediT to specify the contributions of each author in the journal submission system. CRediT replaces the author contribution section, which therefore needs to be removed from the manuscript text. You can use the free text box in our system if you wish to provide more detailed descriptions.

- The information on funding provided in the manuscript and the online submission system needs to be congruent. Currently only DFG is added to the online system; perhaps also medical faculty of the University of Münster, the joint graduate school of the Cells-in-Motion Cluster of Excellence (EXC 1003 - CiM), Germany and the International Max Planck Research School - Molecular Biomedicine, Münste should be added as separate funders in the system.

- Please address the following points in the figure legends:
1) Please provide the exact p values in the legends of figures 1C, 2D, F; 5B, C, D, E; 6D, E; EV1 A, EV2 D, F; EV4 B, C, F, G.
2) Please indicate the statistical test used for data analysis in the legends of figures 1E, EV2 A
3) Please note that the box plots need to be defined in terms of minima, maxima, centre, bounds of box and whiskers, and percentile in the legends of figures 2D, F; 6D, E; EV2D, F; EV4 F, G.
4) Please note that information related to n is missing in the legend of figure 1E

- Suzawa et al, 2025 and Evans et al, 2021 are correctly tagged as [PREPRINT] in the reference list but also need a tag in the manusrcipt text: (preprint: Suzawa et al, 2020) and (preprint: Evans et al, 2021).

- I might have missed it but I couldn't find information on the authority granting ethics approval (Author Checklist line 95) and a description of randomization or similar procedures (Checklist line 81) in the methods section. Please add this information.

- As a standard procedure we edit the abstract. In this case, I tried to reduce redundancy a bit. Please find my suggestion below my signature.

- Finally, EMBO Reports papers are accompanied online by
A) a short (1-2 sentences) summary of the findings and their significance,
B) 2-3 bullet points highlighting key results and
C) a schematic summary figure that provides a sketch of the major findings (not a data image).
Please provide the summary figure as a separate file in PNG or JPG format at a size of 550x300-600 pixels (width x height). Please note that the size is rather small and that text needs to be readable at the final size. Please send us this information along with the revised manuscript.

I look forward to seeing a new revised version of your manuscript as soon as possible.

With kind regards,

Martina Rembold, PhD
Senior Editor
EMBO reports

=================

Referee #1:

Title: A Nanos3-containing protein complex activates RNA translation in primordial germ cells in vivo
Several of my concerns have been minimized in this resubmission. Not the major one though.
Is this ms reporting a candidate-based RNA translation or discovery-based RNA translation? The ms title suggests discovery, and the revelation of new candidates for Nanos regulation - but I am only seeing a candidate responding to Nanos3 activation of RNA-translation? The other two Reviewers have similar comments. Either change the title of the ms to reflect a focused candidate of Nanos3 dependence and/or test/show the breadth of Nanos3 translational regulation. The title is otherwise misleading.
I really like the work and don't want others to be disappointed or misled.

Referee #3:

I thank the authors for their careful revision of the manuscript and for their detailed responses to the reviewers' comments. The revised version of the manuscript has been substantially improved. In particular, the authors have added new experiments, clarified several methodological points, and corrected issues related to figure presentation.

This study investigates the molecular mechanisms underlying the maintenance of primordial germ cell (PGC) identity in zebrafish and proposes that a Nanos3-Dnd1 complex promotes the translation of specific germline RNAs through interaction with the translation initiation factor eIF3d. The work identifies a previously unappreciated functional interaction between Nanos3 and Dnd1 and provides new insights into how germline identity may be stabilized in early PGCs.

The revised manuscript addresses most of the concerns raised in the initial review. The additional experiments, including proximity labeling and RNA immunoprecipitation analyses, strengthen the evidence supporting the interaction between the relevant factors and their association with target RNAs. The clarification of imaging analyses and the correction of figure presentation also improve the overall clarity of the study.

While some aspects of the proposed mechanism remain to be fully established, particularly regarding the precise role of the Nanos3-Dnd1 complex in translation regulation, I believe that the current data sufficiently support the main observations presented in this study. In particular, the identification of the Nanos3-Dnd1 interaction and its role in maintaining germ cell identity represents an interesting and potentially important finding.

Given the scope of EMBO Reports, which often emphasizes novel biological observations and conceptual advances, I believe that the study provides sufficient new insight to merit publication.

Therefore, I support publication of the manuscript after minor revisions addressing the following points.

Minor comments
1. The manuscript proposes that the Nanos3-Dnd1 complex promotes translation through interaction with eIF3d. Although the data are suggestive, the mechanistic interpretation should be described with appropriate caution in the text. Moderating some statements regarding direct translation activation would help avoid overinterpretation.
2. The discussion could benefit from a clearer consideration of previously proposed functions of Dnd1, particularly its role in protecting target mRNAs from miRNA-mediated repression. Integrating this literature would help place the present findings in a broader context.

================

Abstract

Maintaining the germline fate requires tight post-transcriptional control of RNA function. Here, we investigate how primordial germ cell (PGC) identity is maintained in zebrafish and reveal that the conserved RNA-binding proteins Nanos3 and Dead End1 form a complex that safeguards PGC identity. Using transcriptomics and in vivo imaging-based analyses, we show that this complex controls the translational activation and localization of both nanos3 and dead end1 RNAs, establishing a positive feedback loop crucial for regulating their protein expression. These findings uncover a previously unknown layer of control over germline development, where a complex containing Nanos3, a protein associated with the inhibition of RNA translation, acts as an activator by interacting with an eIF3 complex protein to promote translation, thereby maintaining specific RNAs at the periphery of phase-separated germ cell granules. Disrupting the physical interaction between Nanos3 and Dead End1 leads to transdifferentiation of germ cells into somatic lineages. Overall, our findings identify a self-sustaining mechanism of translational activation in vivo, positioning the Nanos3-Dead End1 complex as a central effector of germline fate.

Dear Martina,

Thanks for the comments on the revised version.

We have incorporated all the issues raised and explained within the text what was done for each issue.

- The manuscript sections should be in the following order: Title page - Abstract & Keywords - Introduction - Results - Discussion - Methods - Data Availability - Acknowledgments - Disclosure Statement & Competing Interests - References - Figure Legends - (Main Tables with legends if applicable) - Expanded View Figure Legends.

The manuscript has been reorganized to follow the required section order.

- Declaration of Interests needs to be renamed to Disclosure and Competing Interests Statement.

This section has been renamed to Disclosure and Competing Interests Statement.

- Tables S1 and S2 in the Appendix are complex datasets. Please upload them individually as .xls files called Dataset EV#. The legend is part of the .xls file and placed in a separate tab. Please update all callouts in the paper accordingly. The Appendix is thus obsolete as the movie legend is part of the movie zip file. See next point.

Tables S1 and S2 have been renamed Dataset EV1 and Dataset EV2, and all corresponding mentions of them in the manuscript text have been updated accordingly. The movie legend is now provided as a separate text file included in the zipped Movie EV1 file.

- You state in the methods section that antibodies are listed in Table S3. I think this should refer to the Reagents and Tools table.

The text has been updated to refer to the Reagents and Tools table.

- Table S4 oligonucleotides should be Table EV1. Please update the table name, the legend and the callout in the Reagents and Tools table.

(With this you should have Dataset EV1 and EV2, I believe, Table EV1, Reagents and Tools table).

The datasets and tables have been renamed accordingly. Dataset EV1, Dataset EV2, Table EV1 and the Reagents and Tools table have been uploaded again.

- Movie: the legend should be removed from the manuscript and provided as a text file and then zipped up with the movie; the correct nomenclature should be Movie EV1 in all places (source file names, titles in the system, legend, callout in the manuscript).

We now provide a zipped file labelled Movie EV1 containing the correctly labelled Movie EV1 and its legend as a separate pdf. The manuscript text has been revised accordingly.

- The Data availability section should only refer to the deposited data. Please remove the statement "Further information or requests for resources and reagents should be directed to the Lead Contact, Erez Raz." Moreover, we would need an URL that resolves to the dataset PRJNA1357867.

The Data Availability section has been revised accordingly. The stated sentence has been removed, and a URL resolving to the dataset PRJNA1357867 has been provided.

- The code on github can also be listed in the Data availability section (The code is available on GitHub (https://github.com/Julian-We/PPInet).
The Data Availability section has been updated to include the link to the code.

- The Nomenclature of EV figure legends needs to be Figure EV1, etc. instead of Expanded View EV1.

The manuscript figure legend titles have been revised accordingly.

- Regarding the Author Contributions, we now use CRediT to specify the contributions of each author in the journal submission system. CRediT replaces the author contribution section, which therefore needs to be removed from the manuscript text. You can use the free text box in our system if you wish to provide more detailed descriptions.
The author contributions section has been removed from the manuscript text, and this information is pasted in the Journal submission system.

- The information on funding provided in the manuscript and the online submission system needs to be congruent. Currently only DFG is added to the online system; perhaps also medical faculty of the University of Münster, the joint graduate school of the Cells-in-Motion Cluster of Excellence (EXC 1003 - CiM), Germany and the International Max Planck Research School - Molecular Biomedicine, Münste should be added as separate funders in the system.

The additional funding sources have been added to the online submission system.

- Please address the following points in the figure legends:
1) Please provide the exact p values in the legends of figures 1C, 2D, F; 5B, C, D, E; 6D, E; EV1 A, EV2 D, F; EV4 B, C, F, G.

The indicated graphs were analyzed using the Mann–Whitney U test in Prism. For these comparisons, Prism reports very small values as $P < 0.0001$, which we have indicated in the

figure legends accordingly.

2) Please indicate the statistical test used for data analysis in the legends of figures 1E, EV2 A

We thank the editor for this comment. These analyses are based on bulk RNA-seq data analyzed using DESeq2. The figure legends have been updated to clarify this.

3) Please note that the box plots need to be defined in terms of minima, maxima, centre, bounds of box and whiskers, and percentile in the legends of figures 2D, F; 6D, E; EV2D, F; EV4 F, G.

The figure legends have been updated to define the box plots accordingly.

4) Please note that information related to n is missing in the legend of figure 1E

The figure legends have been updated with n=3 independent biological replicates accordingly.

- Suzawa et al, 2025 and Evans et al, 2021 are correctly tagged as [PREPRINT] in the reference list but also need a tag in the manusrcipt text: (preprint: Suzawa et al, 2020) and (preprint: Evans et al, 2021).

The in-text citations have been updated accordingly to include the preprint tags.

- I might have missed it but I couldn't find information on the authority granting ethics approval (Author Checklist line 95) and a description of randomization or similar procedures (Checklist line 81) in the methods section. Please add this information.

This information can been found in the Methods section. Under Zebrafish husbandry and embryo collection, it is stated that "all experiments and fish maintenance were performed following the regulations of the North Rhine-Westphalian Office of Nature, Environment and Consumer Protection (LANUV NRW)." In addition, under Image acquisition and processing, it is now stated that "all experiments were performed using independent biological replicates and, where applicable, repeated technical replicates to ensure reproducibility."

- As a standard procedure we edit the abstract. In this case, I tried to reduce redundancy a bit. Please find my suggestion below my signature.

We have replaced the submitted abstract with the updated version provided.

- Finally, EMBO Reports papers are accompanied online by
A) a short (1-2 sentences) summary of the findings and their significance,
B) 2-3 bullet points highlighting key results and
C) a schematic summary figure that provides a sketch of the major findings (not a data image).
Please provide the summary figure as a separate file in PNG or JPG format at a size of 550x300-600 pixels (width x height). Please note that the size is rather small and that text

needs to be readable at the final size. Please send us this information along with the revised manuscript.

The summary text has been provided as a separate PDF file (file name "Synopsis"), and the summary figure as a PNG has been uploaded as well.

The referees raised two issues that they recommend changing-

Referee #1:

Is this ms reporting a candidate-based RNA translation or discovery-based RNA translation? The ms title suggests discovery, and the revelation of new candidates for Nanos regulation - but I am only seeing a candidate responding to Nanos3 activation of RNA-translation? The other two Reviewers have similar comments. Either change the title of the ms to reflect a focused candidate of Nanos3 dependence and/or test/show the breadth of Nanos3 translational regulation. The title is otherwise misleading. I really like the work and don't want others to be disappointed or misled.

As suggested, we have changed the title of the paper from-

A Nanos3-containing protein complex activates RNA translation in primordial germ cells in vivo

to-

A Nanos3-containing protein complex can activate RNA translation in primordial germ cells in vivo

The new title does not convey the impression of a genome-wide screen, in which we identified a large/full set of regulated RNAs. Rather, the modified title indicates that we identify a role for Nanos3 in translational activation *in vivo*, with the "can" indicating that we demonstrate the principle using some transcripts.

Referee #3:

Minor comments

1. The manuscript proposes that the Nanos3-Dnd1 complex promotes translation through interaction with eIF3d. Although the data are suggestive, the mechanistic interpretation should be described with appropriate caution in the text. Moderating some statements regarding direct translation activation would help avoid overinterpretation.

To address this point, we have added the following statement to the Discussion: “While our results are consistent with a direct role for the Nanos3–Dnd1–eIF3d complex in activating translation, this point could be further supported by in vitro experiments in which the function of the complex can be tested in isolation.”

2. The discussion could benefit from a clearer consideration of previously proposed functions of Dnd1, particularly its role in protecting target mRNAs from miRNA-mediated repression. Integrating this literature would help place the present findings in a broader context.

We have revised the Discussion to better integrate the functions of Dnd1 we have previously described and include the following statement: “Dnd1 has been reported to promote protein production by enhancing RNA stability and translation through antagonizing miRNA-mediated repression (Kedde et al, 2007). Our findings suggest that, when in complex with Dnd1, in addition to the translation initiation activation, Nanos3 could function in antagonizing miRNA function as well.”

2nd Revision - Editorial Decision 1st Apr 2026

Prof. Erez Raz
University of Münster
Institute for Cell Biology
von Esmarchstr 56
Münster 48149
Germany

Dear Erez,

I am very pleased to accept your manuscript for publication in the next available issue of EMBO reports. Thank you for your contribution to our journal.

Your manuscript will be processed for publication by EMBO Press. It will be copy edited and you will receive page proofs prior to publication. Please note that you will be contacted by Springer Nature Author Services to complete licensing and payment information.

You may qualify for financial assistance for your publication charges - either via a Springer Nature fully open access agreement or an EMBO initiative. Check your eligibility: https://link.springer.com/journal/44319/how-to-publish-with-us

Should you be planning a Press Release on your article, please get in contact with embo_production@springernature.com as early as possible in order to coordinate publication and release dates.

If you have any questions, please do not hesitate to contact the Editorial Office. Thank you for your contribution to EMBO Reports.

Kind regards,

Martina

Martina Rembold, PhD
Senior Editor
EMBO reports

>>> Please note that it is EMBO Reports policy for the transcript of the editorial process (containing referee reports and your response letter) to be published as an online supplement to each paper. If you do NOT want this, you will need to inform the Editorial Office via email immediately. More information is available here: https://link.springer.com/partners/embo-press/editorial-policies#Peer%20review

EMBO Press Author Checklist

Corresponding Author Name: Erez Raz	
Journal Submitted to: EMBO Reports	
Manuscript Number: EMBOR-2025-62376V1	

USEFUL LINKS FOR COMPLETING THIS FORM
[The EMBO Journal - Author Guidelines]
[EMBO Reports - Author Guidelines]
[Molecular Systems Biology - Author Guidelines]
[EMBO Molecular Medicine - Author Guidelines]

Reporting Checklist for Life Science Articles (updated January
This checklist is adapted from Materials Design Analysis Reporting (MDAR) Checklist for Authors. MDAR establishes a minimum set of requirements in transparent reporting in the life sciences (see Statement of Task: 10.31222/osf.io/9sm4x). Please follow the journal's guidelines in preparing your
Please note that a copy of this checklist will be published alongside your article.

Abridged guidelines for figures
1. Data
The data shown in figures should satisfy the following conditions:
- ➞ the data were obtained and processed according to the field's best practice and are presented to reflect the results of the experiments in an accurate and unbiased manner.
- ➞ ideally, figure panels should include only measurements that are directly comparable to each other and obtained with the same assay.
- ➞ plots include clearly labeled error bars for independent experiments and sample sizes. Unless justified, error bars should not be shown for technical
- ➞ if n<5, the individual data points from each experiment should be plotted. Any statistical test employed should be justified.
- ➞ Source Data should be included to report the data underlying figures according to the guidelines set out in the authorship guidelines on Data

2. Captions
Each figure caption should contain the following information, for each panel where they are relevant:
- ➞ a specification of the experimental system investigated (eg cell line, species name).
- ➞ the assay(s) and method(s) used to carry out the reported observations and measurements.
- ➞ an explicit mention of the biological and chemical entity(ies) that are being measured.
- ➞ an explicit mention of the biological and chemical entity(ies) that are altered/varied/perturbed in a controlled manner.
- ➞ the exact sample size (n) for each experimental group/condition, given as a number, not a range;
- ➞ a description of the sample collection allowing the reader to understand whether the samples represent technical or biological replicates (including how many animals, litters, cultures, etc.).
- ➞ a statement of how many times the experiment shown was independently replicated in the laboratory.
- ➞ definitions of statistical methods and measures:
  - common tests, such as t-test (please specify whether paired vs. unpaired), simple χ2 tests, Wilcoxon and Mann-Whitney tests, can be unambiguously identified by name only, but more complex techniques should be described in the methods section;
  - are tests one-sided or two-sided?
  - are there adjustments for multiple comparisons?
  - exact statistical test results, e.g., P values = x but not P values < x;
  - definition of 'center values' as median or average;
  - definition of error bars as s.d. or s.e.m.

Please complete ALL of the questions below.
Select "Not Applicable" only when the requested information is not relevant for your study.

Materials

Newly Created Materials	Information included in the manuscript?	In which section is the information available? (Reagents and Tools Table, Materials and Methods, Figures, Data Availability Section)
New materials and reagents need to be available; do any restrictions apply?	Not Applicable	

Antibodies	Information included in the manuscript?	In which section is the information available? (Reagents and Tools Table, Materials and Methods, Figures, Data Availability Section)
For **antibodies** provide the following information:<br>- Commercial antibodies: RRID (if possible) or supplier name, catalogue number and or/clone number<br>- Non-commercial: RRID or citation	Yes	Reagents and Tools table

DNA and RNA sequences	Information included in the manuscript?	In which section is the information available? (Reagents and Tools Table, Materials and Methods, Figures, Data Availability Section)
**Short novel DNA or RNA including primers, probes**: provide the sequences.	Yes	Table S4_Oligonucleotides

Cell materials	Information included in the manuscript?	In which section is the information available? (Reagents and Tools Table, Materials and Methods, Figures, Data Availability Section)
**Cell lines:** Provide species information, strain. Provide accession number in repository **OR** supplier name, catalog number, clone number, and/**OR** RRID.	Not Applicable	
**Primary cultures:** Provide species, strain, sex of origin, genetic modification status.	Not Applicable	
Report if the cell lines were recently **authenticated** (e.g., by STR profiling) and tested for mycoplasma contamination.	Not Applicable	

Experimental animals	Information included in the manuscript?	In which section is the information available? (Reagents and Tools Table, Materials and Methods, Figures, Data Availability Section)
**Laboratory animals or Model organisms:** Provide species, strain, sex, age, genetic modification status. Provide accession number in repository **OR** supplier name, catalog number, clone number, **OR** RRID.	Yes	Materials and methods
**Animal observed in or captured from the field:** Provide species, sex, and age where possible.	Not Applicable	
Please detail **housing and husbandry conditions**.	Yes	Materials and methods

Plants and microbes	Information included in the manuscript?	In which section is the information available? (Reagents and Tools Table, Materials and Methods, Figures, Data Availability Section)
**Plants:** provide species and strain, ecotype and cultivar where relevant, unique accession number if available, and source (including location for collected wild specimens).	Not Applicable	
**Microbes:** provide species and strain, unique accession number if available, and source.	Not Applicable	

Human research participants	Information included in the manuscript?	In which section is the information available? (Reagents and Tools Table, Materials and Methods, Figures, Data Availability Section)
If collected and within the bounds of privacy constraints report on age, sex and gender or ethnicity for all study participants.	Not Applicable	

Core facilities	Information included in the manuscript?	In which section is the information available? (Reagents and Tools Table, Materials and Methods, Figures, Data Availability Section)
If your work benefited from core facilities, was their service mentioned in the acknowledgments section?	Not Applicable	

Design

Study protocol	Information included in the manuscript?	In which section is the information available? (Reagents and Tools Table, Materials and Methods, Figures, Data Availability Section)
If study protocol has been **pre-registered, provide DOI in the manuscript**. For clinical trials, provide the trial registration number **OR** cite DOI.	Not Applicable	
Report the **clinical trial registration number** (at ClinicalTrials.gov or equivalent), where applicable.	Not Applicable	

Laboratory protocol	Information included in the manuscript?	In which section is the information available? (Reagents and Tools Table, Materials and Methods, Figures, Data Availability Section)
Provide DOI OR other citation details if **external detailed step-by-step protocols** are available.	Not Applicable	

Experimental study design and statistics	Information included in the manuscript?	In which section is the information available? (Reagents and Tools Table, Materials and Methods, Figures, Data Availability Section)
Include a statement about **sample size** estimate even if no statistical methods were used.	Yes	Figure legends
Were any steps taken to minimize the effects of subjective bias when allocating animals/samples to treatment (e.g. **randomization procedure**)? If yes, have they been described?	Yes	Materials and methods
Include a statement about **blinding** even if no blinding was done.	Not Applicable	
Describe **inclusion/exclusion criteria** if samples or animals were excluded from the analysis. Were the criteria pre-established?<br><br>If sample or data points were omitted from analysis, report if this was due to attrition or intentional exclusion and provide justification.	Not Applicable	
For every figure, are **statistical tests** justified as appropriate? Do the data meet the assumptions of the tests (e.g., normal distribution)? Describe any methods used to assess it. Is there an estimate of variation within each group of data? Is the variance similar between the groups that are being statistically compared?	Yes	Materials and methods and Figure legends

Sample definition and in-laboratory replication	Information included in the manuscript?	In which section is the information available? (Reagents and Tools Table, Materials and Methods, Figures, Data Availability Section)
In the figure legends: state number of times the experiment was **replicated** in laboratory.	Yes	Figure legends
In the figure legends: define whether data describe **technical or biological replicates**.	Yes	Figure legends

Ethics

Ethics	Information included in the manuscript?	In which section is the information available? (Reagents and Tools Table, Materials and Methods, Figures, Data Availability Section)
Studies involving **human participants**: State details of **authority granting ethics approval** (IRB or equivalent committee(s), provide reference number for approval.	Not Applicable	
Studies involving **human participants**: Include a statement confirming that **informed consent** was obtained from all subjects and that the experiments conformed to the principles set out in the WMA Declaration of Helsinki and the Department of Health and Human Services Belmont Report.	Not Applicable	
Studies involving **human participants:** For publication of **patient photos**, include a statement confirming that consent to publish was obtained.	Not Applicable	
Studies involving experimental **animals**: State details of **authority granting ethics approval** (IRB or equivalent committee(s), provide reference number for approval. Include a statement of compliance with ethical regulations.	Yes	Materials and methods
Studies involving **specimen and field samples:** State if relevant **permits** obtained, provide details of authority approving study; if none were required, explain why.	Not Applicable	

Dual Use Research of Concern (DURC)	Information included in the manuscript?	In which section is the information available? (Reagents and Tools Table, Materials and Methods, Figures, Data Availability Section)
Could your study fall under dual use research restrictions? Please check biosecurity documents and list of **select agents and toxins** (CDC): https://www.selectagents.gov/sat/list.htm	Not Applicable	
If you used a select agent, is the security level of the lab appropriate and reported in the manuscript?	Not Applicable	
If a study is subject to dual use research of concern regulations, is the name of the **authority granting approval and reference number** for the regulatory approval provided in the manuscript?	Not Applicable	

Reporting

The MDAR framework recommends adoption of discipline-specific guidelines, established and endorsed through community initiatives. Journals have their own policy about requiring specific guidelines and recommendations to complement MDAR.

Adherence to community standards	Information included in the manuscript?	In which section is the information available? (Reagents and Tools Table, Materials and Methods, Figures, Data Availability Section)
State if relevant guidelines or checklists (e.g., **ICMJE, MIBBI, ARRIVE, PRISMA**) have been followed or provided.	Not Applicable	
For **tumor marker prognostic studies,** we recommend that you follow the **REMARK** reporting guidelines (see link list at top right). See author guidelines, under 'Reporting Guidelines'. Please confirm you have followed these guidelines.	Not Applicable	
For **phase II and III randomized controlled trials**, please refer to the **CONSORT** flow diagram (see link list at top right) and submit the CONSORT checklist (see link list at top right) with your submission. See author guidelines, under 'Reporting Guidelines'. Please confirm you have submitted this list.	Not Applicable	

Data Availability

Data availability	Information included in the manuscript?	In which section is the information available? (Reagents and Tools Table, Materials and Methods, Figures, Data Availability Section)
Have **primary datasets** been deposited according to the journal's guidelines (see 'Data Deposition' section) and the respective accession numbers provided in the Data Availability Section?	Yes	Data Availability Section
Were **human clinical and genomic datasets** deposited in a public access-controlled repository in accordance to ethical obligations to the patients and to the applicable consent agreement?	Not Applicable	
Are **computational models** that are central and integral to a study available without restrictions in a machine-readable form? Were the relevant accession numbers or links provided?	Not Applicable	
If publicly available data were reused, provide the respective **data citations in the reference list**.	Not Applicable	
